# The aspartyl protease DDI2 drives adaptation to proteasome inhibition in multiple myeloma

**DOI:** 10.1038/s41419-022-04925-3

**Published:** 2022-05-19

**Authors:** Mélanie Op, Sérgio T. Ribeiro, Claire Chavarria, Aude De Gassart, Léa Zaffalon, Fabio Martinon

**Affiliations:** 1 Department of Immunobiology, University of Lausanne, 155 Ch. des Boveresses, 1066 Epalinges, Switzerland; 2 Present Address: ImCheck Therapeutics, 13009 Marseille, France

**Keywords:** Myeloma, Proteasome, Stress signalling

## Abstract

Proteasome inhibitors, such as bortezomib, are first-line therapy against multiple myeloma (MM). Unfortunately, patients frequently become refractory to this treatment. The transcription factor NRF1 has been proposed to initiate an adaptation program that regulates proteasome levels. In the context of proteasome inhibition, the cytosolic protease DDI2 cleaves NRF1 to release an active fragment that translocates to the nucleus to promote the transcription of new proteasome subunits. However, the contribution of the DDI2-NRF1 pathway to bortezomib resistance is poorly understood. Here we show that upon prolonged bortezomib treatment, MM cells become resistant to proteasome inhibition by increasing the expression of DDI2 and consequently activation of NRF1. Furthermore, we found that many MM cells became more sensitive to proteasome impairment in the context of DDI2 deficiency. Mechanistically, we demonstrate that both the protease and the HDD domains of DDI2 are required to activate NRF1. Finally, we show that partial inhibition of the DDI2-protease domain with the antiviral drug nelfinavir increased bortezomib susceptibility in treated MM cells. Altogether, these findings define the DDI2-NRF1 pathway as an essential program contributing to proteasome inhibition responses and identifying DDI2 domains that could be targets of interest in bortezomib-treated MM patients.

## Introduction

Multiple myeloma (MM) is a plasma cell cancer representing the second most common hematologic malignancy in Western countries [1, 2]. Plasma cells are terminally differentiated B-lineage lymphocytes that secrete large amounts of immunoglobulins. Previous studies suggested that each plasma cell can secrete the equivalent of its mass in immunoglobulins, overloading the translation, folding, and secretory capacity of the cell [3]. Consequently, to maintain cellular proteostasis and survival, secretory plasma cell malignancies rely on adaptation programs and stress response pathways [4]. The Ubiquitin-Proteasome System plays a crucial role in proteostasis by degrading misfolded proteins [5]. Alteration of the proteostasis network may explain why proteasome inhibitors (PI) decrease the viability of MM cells and significantly improve the prognosis of MM patients [6, 7].

Since the FDA approved the PI bortezomib (BTZ) for the treatment of MM and mantle cell lymphoma (MCL) in 2003, clinical evidence showed that targeting the catalytic activity of the proteasome was a breakthrough in the treatment of these cancers [8, 9]. Unfortunately, drug resistance is a significant drawback in PI therapy, leading to relapses in MM patients [10]. Acquired resistance to BTZ is complex, multifactorial, and poorly understood. It includes overexpression of efflux pumps, mutations within BTZ’s target PSMB5, and the induction of compensatory proteolytic pathways [11].

Recently the Nuclear Factor, Erythroid 2 Like 1 (NFE2L1) transcription factor, commonly known as NRF1, has been shown to contribute to the maintenance of proteasome function [12–14]. The mechanisms of activation of NRF1 are atypical. Under basal conditions, NRF1 is localized in the endoplasmic reticulum (ER) and undergoes translocation and degradation in the cytosol. In the context of impaired proteasome activity, NRF1 degradation is decreased. As a result, part of the NRF1 pool that reaches the cytosol undergoes post-translational modifications that promote its nuclear translocation and transcriptional activity leading to the expression of proteasome subunits [12, 15, 16]. These findings support a model in which the amount of proteasome activity is regulated by an adaptation program induced by transcriptionally active NRF1.

The mechanisms controlling the activation of NRF1 are still poorly understood. Within the ER, NRF1 undergoes N-glycosylation. Then, NRF1 is targeted to the ERAD machinery for retrotranslocation in the cytosol where it undergoes a series of modifications that contributes to its activation. Deglycosylation by NGLY1 was shown to be required for NRF1 activity [17]. In C. elegans, deglycosylation of NRF1 homolog (SKN-1) by PNG-1 is coupled with the editing of N-glycosylated asparagine residues to aspartic acid. This post-translational change of the amino acid sequence is required for maximal transcriptional activity [18].

Proteolytic processing of NRF1 in the cytosol by the aspartyl protease DDI2 is another crucial mechanism that regulates NRF1 activity [19]. Alterations of DDI2 functions were found to potentiate the cytotoxicity of PI in a triple-negative breast cancer model, indicating that this protease could interfere with clinical responses to proteasome inhibition [20]. However, DDI2 is still a poorly understood cytosolic protease, whose only substrate identified to date is NRF1.

In this study, we investigated the contribution of the DDI2-NRF1 pathway to BTZ-mediated toxicity and in the course of drug resistance acquisition in MM. We demonstrated that DDI2 plays an essential role in response to treatment with BTZ in MM cells. Activation of NRF1 by DDI2 contributes to the mechanisms driving BTZ resistance by initiating a proteasome bounce-back response that confers cell proteostasis. Interestingly, we found that nelfinavir, a drug designed to target the HIV protease, partially decreased the activity of DDI2 and potentiated BTZ efficacy in MM. Our study indicates that the development of specific DDI2 inhibitors, in combination with PI, may present new therapeutic opportunities in MM.

## Materials and methods

### Cell culture

ARH77, U266, and L363 cell lines were provided by Prof. Pascal Schneider, University of Lausanne, Switzerland. AMO-1, and RPMI8226 cell lines were provided by Dr. Lenka Besse, Kantonsspital St. Gallen, Switzerland. MM.1 S cells were purchased from ATCC (CRL-2974™). All those cell lines were cultured in RPMI 1640 supplemented with 10% fetal bovine serum (FBS). Cells were tested regularly for mycoplasma contamination. Cell Line Authentication was performed for the cells used in the study using highly-polymorphic short tandem repeat loci (STRs) (Microsynth).

### Generation of stable cell lines

LentiCRISPR-v2 for DDI2 and NRF1 targeting: Optimized CRISPR target sequences were cloned into the lentiCRISPR-v2 vector (Addgene#52961). A sequence targeting luciferase was used as a control single guide RNA (sgRNA). The primers are listed in supplementary table 1.

Crispr/Cas9 lentiviruses were produced as previously describe [21]. Briefly, 40 μl of viral preparation was used to infect the cells. Positive populations were selected with 2–3 μg/ml puromycin for 15 days. To obtain full KO cell lines, populations were cloned by limit dilution. The selected clones are referred to as clX in the figure legends.

The different constructs and RVP-contained proteins were subcloned into the pENTR-1A dual selection vector (Invitrogen) and then insert by LR reaction in a pINDUCER21, a doxycycline-inducible lentiviral vector plasmid. Lentiviruses were produced as previously described [22]. All the constructs have an N-terminal FLAG-tag.

### RNA isolation, reverse transcription, and RT-PCR

Total RNA from cells and tissues was extracted with Trizol (Invitrogen) according to the manufacturer’s protocol. For the reverse transcription, 10 μl of RNA sample diluted in DEPC treated water (0.4–2 μg of RNA) was mixed with 10 μl of 2× Reverse Transcription master mix (Applied Biosystems).

For the quantitative real-time PCR, the SYBR Green fluorescent reagent was used, and the PCR was run on a LightCycler480 Real-Time PCR System from Roche.

### Cytotoxic assay

Multiple myeloma and EBV-positive B cells were plated in 96-well plates at a final concentration of 0.4–0.6 × 10^6^ cells/ml in 80 µl RPMI medium per well. Then 5× concentrated proteasome inhibitor treatments were added in 20 µl of medium making 1× final concentration in the total volume of 100 µl per well. After defined incubation time cell viability was assessed using a 3 - (4,5-dimethylthiazol-2-yl) - 5 - (3-carboxymethoxyphenyl) - 2 - (4-sulfonphenyl) - 2H tetrazolium (MTS) assay (Promega, Madison, WI). Twenty microliters of the MTS reagent was added to each well and the plates were incubated for 2 h at 37 °C. The viability of the samples was estimated by measuring sample absorbance at 492 nm using a spectrophotometric microplate reader. The inhibition of cell proliferation was expressed as the percentage of vehicle control-treated cells.

## Results

### DDI2 contributes to adaptation to Bortezomib treatment in multiple myeloma cells

**Fig. 1:**
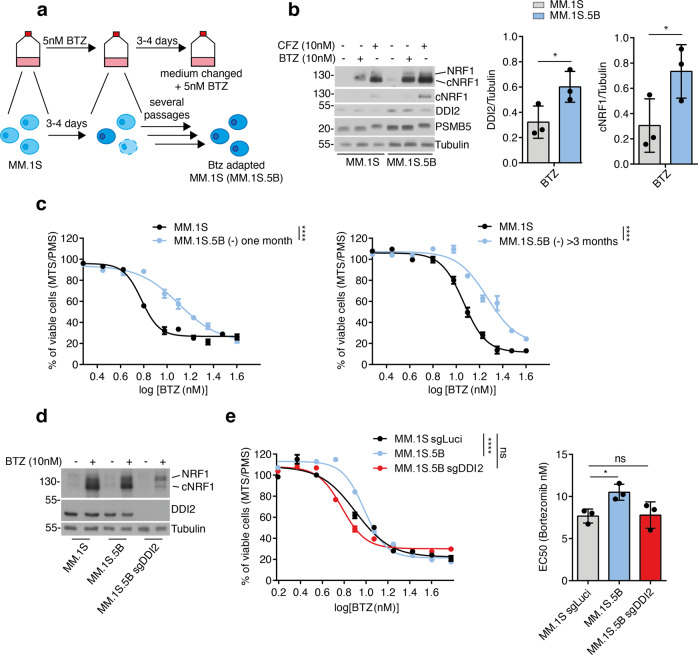
DDI2 deletion restores bortezomib sensitivity in adapted multiple myeloma cells. **a** MM.1 S were cultured for more than three weeks in presence of a 5 nM bortezomib (BTZ). Culture medium is replaced every 3–4 days. For further analysis adapted MM.1 S.5B cells were cultured in absence of bortezomib for more than 48 h, before analysis. **b** Adapted MM.1 S.5B cells and parental cells were treated with vehicle or 10 nM of proteasome inhibitors BTZ or carfilzomib (CFZ) as indicated. Protein levels of NRF1, DDI2, and PSMB5 were analyzed by immunoblotting, tubulin is used as a loading control, cNRF1, indicates cleaved NRF1. DDI2 and cNRF1 were quantified using Image J (right panel), *P*-values were calculated using two-tailed unpaired Student’s *t*-tests (left panel). **c** Viability assay of parental cells and MM.1 S.5B cultured 3 weeks or 3 months without BTZ treatment. *P*-values were calculated using two-way ANOVA. **d**, **e** Adapted MM.1 S.5B expressing a DDI2 sgRNA (MM.1 S.5B sgDDI2) were generated. Expression of DDI2 and NRF1 cleavage upon treatment with BTZ was analyzed by immunoblotting (**d**). Bortezomib sensitivity of the luciferase control and DDI2-deficient population was assessed by viability assay (**e**, left panel), Dots (bar) graph represents the EC50 (half-maximal effective concentration) of the dose responses, data are from three independent experiments performed in triplicate (**e**, right panel). *P*-values were calculated using one-way ANOVA followed by Dunnett’s multiple comparison tests.

To study the resistance mechanisms of multiple myeloma (MM) to PI treatments, we generated a resistant cell line model based on MM.1 S cells [23]. These cells have a high proteasomal workload compared to other MM, making them particularly sensitive to BTZ treatments [24, 25]. To generate a PI-resistant cell population, we cultured MM.1 S cells with a sub-lethal concentration of BTZ (Fig. 1a). After 4 weeks of BTZ treatment, adapted cells were characterized 48 h after treatment withdrawal.

When we analyzed the NRF1-DDI2 pathways, we found that compared to parental cells, adapted MM.1 S.5B cells showed increased expression of DDI2 and increased cleavage of NRF1 upon treatment with the PIs BTZ or Carfilzomib (CFZ) (Fig. 1b). Moreover, adapted cells were less sensitive to proteasome inhibition compared to parental cells (Fig. 1c). Importantly BTZ resistance was conserved even after 3 months of culture in the absence of BTZ, suggesting the engagement of a stable adaptation response (Fig. 1c, right panel). To interrogate DDI2 contribution to this adaptation response, we used the CRISPR-Cas9 technology to delete DDI2 in adapted MM.1 S.5B cells. In unstressed cells, including MM.1 S.5B cells, we could generate DDI2-deficient populations with similar efficacy as control cells expressing a sgRNA targeting luciferase, suggesting that DDI2 is not an essential protease in multiple myeloma. In contrast, we found that in stressed cells treated with BTZ, DDI2 deletion decreased NRF1 proteolytic maturation (Fig. 1d) and restored complete sensitization to BTZ treatment (Fig. 1e). These findings suggest that DDI2 may contribute to BTZ adaptation in MM cells.

### DDI2 deficiency increases bortezomib sensitivity of several myeloma cell lines

**Fig. 2:**
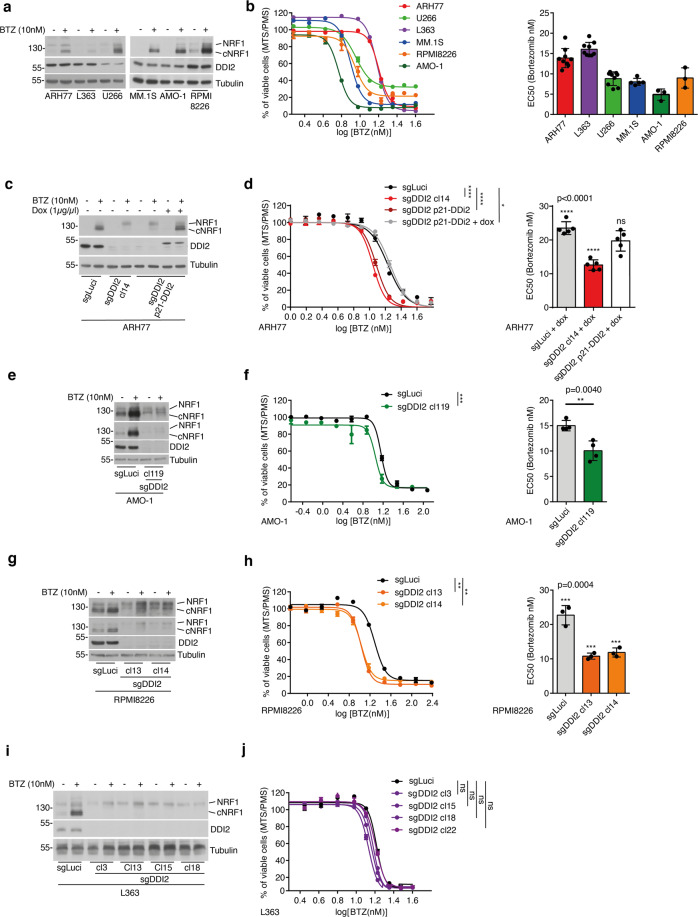
DDI2 deficiency affects Bortezomib sensitivity of sensitive and resistant Myeloma cells. **a**, **b** Indicated multiple myeloma cell lines were treated with BTZ or vehicle and analyzed by immunoblot for protein expression of DDI2 and NRF1, cNRF1, indicates cleaved NRF1. Tubulin is used as a loading control (**a**). Viability assay was assessed by MTS/PMS assay. Dots graph represents the EC50 (half-maximal effective concentration) of the dose responses (**b**). **c**, **d** A representative ARH77 clone expressing DDI2 sgRNA and deficient for DDI2 expression was reconstituted with an inducible N-ter FLAG-DDI2 construct (backbone PINDUCER21) and treated with BTZ and doxycycline (Dox) as indicated. The expression of DDI2, NRF1, and cNRF1 was monitored by immunoblotting (**c**). Viability assay of control cells and reconstituted cells treated with BTZ or Dox was performed as indicated (**d**). **e**–**j** AMO-1, RPMI8226, and L363 populations expressing control luciferase (Luci) sgRNA or representative clonal DDI2-deficient populations were treated with BTZ or vehicle as indicated and analyzed by immunoblotting for expression of DDI2, NRF1, and cNRF1 (**e**, **g**, **i**). Sensitivity to BTZ of control cells and representative DDI2-deficient clones were analyzed by viability assay (**f**, **h**, **j**). Curve graphs are from one representative experiment of two or three replicates. *P*-values were calculated using two-way ANOVA between control cells and KO cells. Dots graph represents the EC50 (half-maximal effective concentration) of the dose responses; data are from at least three independent experiments performed in triplicate. *P*-values were calculated using one-way ANOVA followed by Dunnett’s multiple comparison tests (**d**, **h**) or using two-tailed unpaired Student’s *t*-tests (**f**).

We analyzed several MM cell lines for NRF1 maturation (Fig. 2a). The ARH77 line was established from a patient diagnosed with an IgG Plasma Cell Leukemia (PCL), an advanced stage of MM [26, 27]. We observed that ARH77 were among the less BTZ sensitive cell lines of our panel similar to the L-363, also described as originating from a PCL (Fig. 2b) [28]. Furthermore, we found that in response to BTZ, the myeloma cells tested showed variability of sensitivity and diverse patterns of NRF1 maturation. For example, compared to L-363, ARH77 showed a more robust maturation of NRF1 after proteasome inhibition (Fig. 2a). Altogether, these findings are consistent with the fact that different mechanisms of BTZ resistance can arise in MM and indicate that increased NRF1 activation could be one such mechanism.

Because ARH77 appeared as a model of resistant cell lines with robust NRF1 maturation, we selected this model to investigate DDI2 and NRF1 contribution to BTZ treatment. We used the CRISPR-Cas9 technology to delete DDI2 and NRF1 in these cells (Fig. 2c, Fig. s1a). We observed that DDI2 deficiency increased BTZ sensitivity (Fig. 2d, Fig. s1a), similar to the response seen in NRF1-deficient cells (Fig. s1a). To confirm that the observed phenotype is caused by DDI2 deletion, we reconstituted a representative DDI2-deficient population with a construct expressing a CRISPR-resistant DDI2. Consistently, we found that DDI2 reconstitution restored BTZ resistance in ARH77 (Fig. 2c, d).

To further validate these observations, we next investigated DDI2 involvement in additional cell lines. In line with a recent report [29], several MM lines showed increased sensitivity to BTZ treatment upon DDI2 depletion. We found that AMO-1, an established MM cell line [30], showed increased sensitivity to BTZ treatment upon DDI2 deficiency (Fig. 2e, f). Similarly, we observed that in RPMI8226 cells, which have a solid overall proteasomal activity [24], DDI2 deletion increased BTZ sensitivity (Fig. 2g, h). Moreover, we found that DDI2 deficiency affected BTZ susceptibility in other cell types, including 293T HAP-1 and HeLa cells (Fig. S1b-d). These observations are consistent with observations showing increased susceptibility to proteasome inhibition upon expression of a defective DDI2 protein in the colorectal carcinoma cell line, HCT116 [31], or upon DDI2 deficiency in the triple-negative breast cancer cell line, MDA-MB-231 [20].

In contrast, we found that the viability of L-363 cells, which showed weak NRF1 maturation, was not affected upon BTZ treatment after DDI2 depletion (Fig. 2i, j). These observations suggest that impairing DDI2 activity could decrease BTZ resistance in most but not all MM cells.

### DDI2 contributes to proteasome adaptation by increasing proteasome activity

**Fig. 3:**
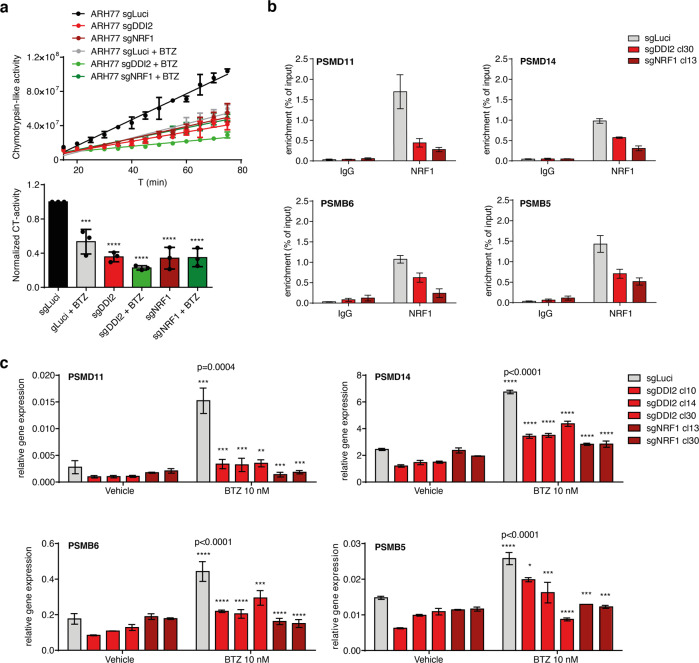
DDI2 deficiency impairs the chymotrypsin-like activity of the proteasome. **a** ARH77 populations expressing sgLuci (control), sgDDI2 (DDI2 deficient), or sgNRF1 (NRF1 deficient) were treated with 100 nM BTZ for 6 h. Chymotrypsin-like activity of the proteasome was measured using fluorescent substrates. Dots (bar) graph represents the chymotrypsin-like (CT) activity of the different groups normalized with the untreated ARH77 expressing sgLuci. Data are from three independent experiments performed in duplicate. Significance was calculated using one-way ANOVA followed by Dunnett’s multiple comparison tests. **b** ChIP was performed on ARH77 sgLuci populations or representative DDI2 and NRF1-deficient clones treated for 24 h with 10 nM BTZ using NRF1-specific antibody or IgG control. Enrichments of indicated proteasome subunit promoters were probed by real-time PCR (mean and SD of technical triplicates of one representative experiment of three). **c** Indicated cells were treated 12 h with 10 nM BTZ and analyzed for proteasome subunits (PSM) mRNA expression by real-time PCR. Normalization was done relative to SRPR levels. *P*-values were calculated using one-way ANOVA followed by Dunnett’s multiple comparison tests.

Because MM cells with high proteasomal activity relied on DDI2 for survival upon treatment with BTZ, we investigated the contribution of the DDI2-NRF1 pathway to proteasome activity at basal and upon treatment with BTZ. We used a Suc-LLVY-AMC fluorogenic peptide substrate that produced fluorescence after degradation to measure the chymotrypsin-like activity of the proteasome. As expected, BTZ treatment decreased the proteasome activity of the control ARH77 population expressing sgLuci (Fig. 3a). Interestingly we found that in clones with DDI2 or NRF1 deficiency, basal proteasome activity was reduced to the same level observed upon BTZ treatment (Fig. 3a). These data indicated that in these cells, DDI2 and NRF1 are required to maintain optimal DDI2-NRF1 pathway, independently of proteasome inhibition.

Then, because BTZ treatment increases proteasome expression, and NRF1 is a regulator of proteasome subunit gene expression, we investigated the contribution of NRF1 and DDI2 to this proteasome bounce-back response in MM. Using chromatin immunoprecipitation, we tested several PSM subunits and found that upon BTZ treatment, NRF1 was recruited to PSMD11, PSMD14, PSMB6, and PSMB5 promoters in a DDI2 dependent manner (Fig. 3b). In addition, BTZ-mediated increase of these proteasome subunits mRNA was reduced in DDI2 and NRF1-deficient clones compared to control populations (Fig. 3c). These observations suggest that the transcriptional activity of the DDI2-NRF1 pathway contributes, at least in part, to the proteasome bounce-back response in ARH77 cells.

### The HDD and RVP domains of DDI2 are required for NRF1 activation

**Fig. 4:**
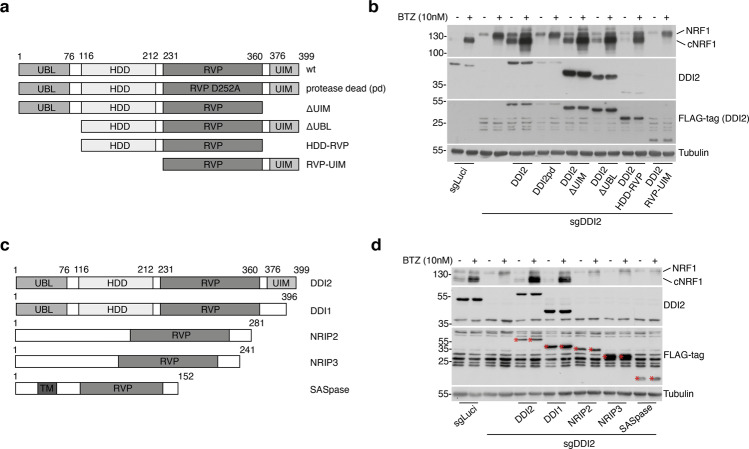
The RVP and HDD domains of DDI2 are essential and sufficient for NRF1 maturation. **a** Schematic diagram of the different doxycycline-inducible and N-ter FLAG-tagged DDI2 constructs used in this study. These constructs were engineered with a silent mutation impairing Crispr/Cas9 cleavage and stably introduced in ARH77 cells deficient for DDI2. **b** Protein expression of NRF1, DDI2, and FLAG was analyzed by immunoblot blot upon treatment with doxycycline, in the presence or absence of BTZ. **c** Schematic diagram of the different doxycycline-inducible and FLAG-tagged constructs expressing known RVP-containing proteases. **d** DDI2-deficient ARH77 cells were stably reconstituted with the constructs depicted in **c**. Cells were treated with doxycycline and BTZ as indicated, and protein expression for NRF1, DDI2, and FLAG-tagged proteins was analyzed by immunoblotting. Tubulin is used as a loading control. The white stars are there to differentiate the FLAG-tagged proteins from the background noise.

While the structure of human DDI2 has been partly described the function of its different domains is still not fully understood [32]. DDI2 harbors a ubiquitin-like (UBL) domain at the N-terminus and a putative ubiquitin interacting motif (UIM) at the C-terminus. The UBL is followed by the helical domain of Ddi1 (HDD), an ~100 amino acid-long region that is conserved among the different Ddi1 orthologs [32]. The retroviral protease-like domain (RVP) harbors the catalytic domain of DDI2 that was proposed to cleave the target proteins [19]. To determine the different DDI2 domains required for NRF1 maturation, we reconstituted the DDI2-deficient ARH77 cells with different versions of DDI2. We used doxycycline-inducible vectors coding for DDI2, a version of DDI2 with a mutation in the active site of the RVP, and a series of truncated DDI2 constructs (Fig. 4a). We analyzed NRF1 maturation following DDI2 expression in the context of BTZ treatment. As expected, we observed that the protease-deficient DDI2 construct could not promote the proteolytic maturation of NRF1. However, the RVP domain coupled with the UIM motif was not sufficient to cleave NRF1 (Fig. 4b). In contrast, the combination of the HDD and the RVP domains of DDI2 fully restored NRF1 maturation and increased PSMB5 expression (Fig. 4b, Fig. s2a). This observation was confirmed in DDI2-deficient HeLa cells transiently reconstituted with DDI2 constructs (Fig. s2b). These findings identify the RVP and the HDD regions as crucial mediators of DDI2 proteolytic function and suggest that the UIM and UBL domains are dispensable for NRF1 activation.

To study DDI2 specificity in recognizing and cleaving NRF1, we interrogated whether other known proteins harboring the RVP fold could compensate for DDI2 deficiency (Fig. 4c). We reconstituted DDI2-deficient ARH77 cells with DDI1, a paralogue of DDI2, NRIP2, and NRIP3, two members of the nuclear receptor-interacting protein group, and SASpase, an RVP-containing protein primarily expressed in the epidermis [33, 34]. We found that the expression of DDI1 could compensate for DDI2 deficiency and mediated NRF1 maturation in the presence of BTZ (Fig. 4d, Fig. s2a). In contrast, RVP-containing proteins that lack the HDD region, including the SASpase and NRIP proteins, could not compensate for DDI2 absence (Fig. 4d).

### The protease inhibitor nelfinavir partially inhibits DDI2 and potentiates BTZ toxicity in MM cells

**Fig. 5:**
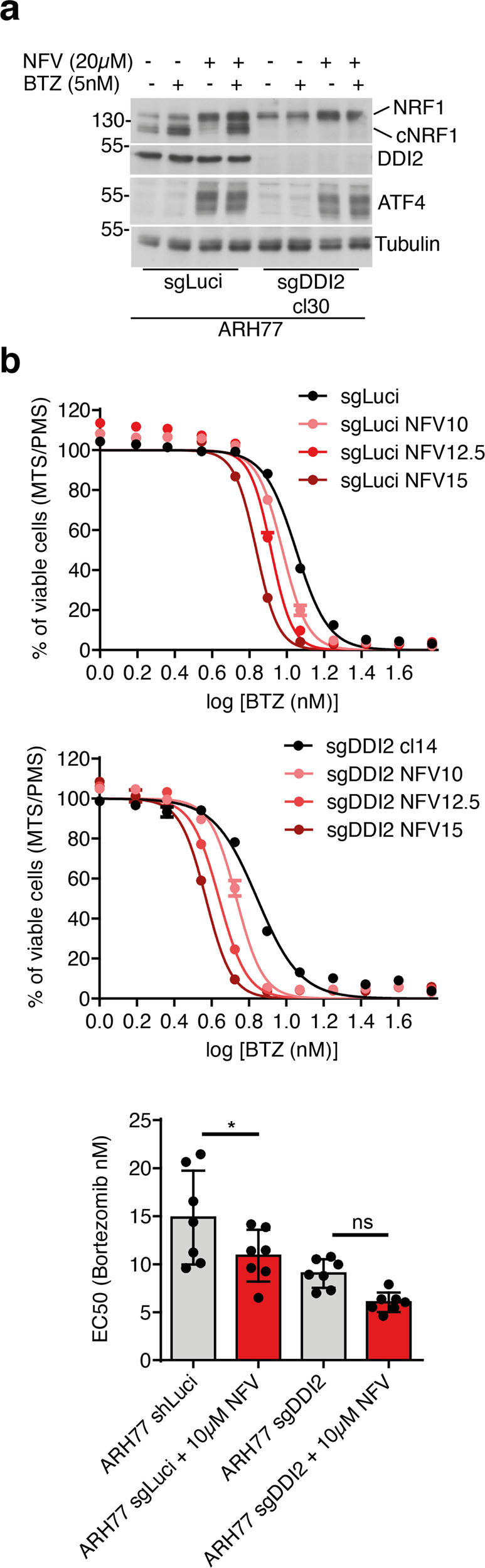
Nelfinavir increased Bortezomib sensitivity. **a, b** ARH77 cells control population, or deficient for DDI2 were treated with BTZ in the presence or absence of Nelfinavir (NFV) as indicated. Protein expression of DDI2, NRF1, and ATF4 was assessed by immunoblot Blot. Tubulin is used as a loading control (**a**). BTZ sensitivity was analyzed by viability assay; curves are from one representative experiment performed in triplicate. Dots graph represents the EC50 (half-maximal effective concentration) of the dose responses; data are from independent experiments performed in triplicate. *P*-values were calculated using one-way ANOVA with Bonferroni multiple comparison post-tests.

Nelfinavir (NFV) is a clinical available antiviral drug initially developed to target the RVP domain of the HIV protease. It has been reported that treatment with nelfinavir has off-target effects in humans that show anti-cancer properties [35, 36]. Several pathways have been proposed to contribute to NFV antitumoral properties, including the induction of ER-stress and the regulation of translation elongation [37, 38]. However, the direct target of NFV in humans has not been identified yet. Recent studies suggested that NFV inhibits DDI2, and may therefore affect MM growth [31, 39]. Because the HIV and DDI2 RVP domains share structural similarities, it has been proposed that NFV may target DDI2 [31]. To test this hypothesis in our model, we challenged the ARH77 cells expressing sgLuci or sgDDI2 with BTZ in the presence or absence of NFV. Consistent with our previous findings, we observed that treatment with NFV activates the Integrated Stress Response (ISR) as measured by ATF4 protein production [21]. Engagement of the ISR is observed in both DDI2 proficient and deficient cells, indicating that DDI2 is not involved in NFV regulation of translation initiation (Fig. 5a). In addition, we observed that in the presence of NFV, basal activation of NRF1 was consistently reduced compared to untreated cells (Fig. 5a). However, in the presence of BTZ, while we observed increased accumulation of uncleaved NRF1 precursor, DDI2-mediated NRF1 maturation was still detected (Fig. 5a). These findings indicate that in the context of robust activation of NRF1, NFV-mediated inhibition of DDI2 cannot abolish its enzymatic activity. To identify better DDI2 inhibitors, we tested several analogs of NFV identified in the NCI Open Chemical Repository Collection [40]. Still, none of these molecules showed a robust impact on the maturation of NRF1 (Fig. s3a). NFR-mediated inhibition of NRF1 was also observed in HeLa, HAP-1, and AMO-1 cells (Fig. s3b–d). NFV was particularly effective in HeLa cells as measured by decreased BTZ-mediated NRF1 cleavage. While expression of DDI2 was required HeLa cells, reconstitution with increasing amounts of DDI2 did not restore NRF1 cleavage in the presence of NFV, further indicating that NFV is particularly effective in this model (Fig. s3e).

To determine if NFV-mediated cytotoxicity potentiates BTZ treatments in MM, we treated ARH77 cells with BTZ in the combination with NFV. We observed that NFV significantly improved the efficacy of BTZ treatment (Fig. 5b). To determine if NFV sensitization relied on DDI2, we treated DDI2-deficient cells with NFV combined with BTZ. In this context, NFV could still increase the sensitivity of DDI2-deficient cells. The statistical significance was decreased compared to the same experiment in DDI2 proficient cells (Fig. 5b). However, the trend was consistent and suggested that while NFV function by decreasing DDI2 proteolytic activity, additional targets contribute to NFV-mediated toxicity in cancer [38].

## Discussion

In this study, we propose that adaptation to treatment with BTZ in MM can rely on a stable increase in the DDI2-NRF1 pathway. The mechanisms involved are still unclear. A possible mechanism may involve epigenetic changes [41]. Like the mechanisms observed in the context of trained immunity [42], proteasome stress may activate long-term functional reprogramming of cells, to maintain proteostasis. Evidence that mechanisms are controlling the amount of the DDI2-NRF1 pathway is also supported by the observation that different MM cell lines have variable protein amounts of the pathway. Some cell lines analyzed in this study, such as the L363, display a relatively weak NRF1 activation. In contrast, other cells have robust NRF1 maturation that is detected in the absence of BTZ treatment, suggesting that some MM may engage the DDI2-NRF1 pathways constitutively, either to respond to a very high demand in proteasome activity or as part of developed mechanisms of resistance. In line with the observed constitutive activation of NRF1, we found that DDI2 deficiency impacted basal proteasomal activity in MM. These cells may, therefore, constitutively engage the DDI2-NRF1 pathway to maintain full proteasome activity. However, despite constitutive engagement of the DDI2-NRF1 pathway, we were able to select and expand DDI2 or NRF1-deficient MM monoclones to the same efficacy as with control populations, suggesting that neither DDI2 nor NRF1 are essential for MM survival in unstressed conditions.

How the DDI2-NRF1 pathway contributes to the maintenance of proteostasis is still unclear. Consistently with other studies [20], we observed a defect in proteasome function in the absence of DDI2. However, decreased expression of proteasome subunits was significant but relatively modest in MM. DDI2 deficiency resulted in less than a two-fold decrease in the transcriptional induction of proteasome subunits. Thus, while these defects may account for the increased BTZ susceptibility, additional pathways engaged by DDI2 may also contribute. DDI2 may directly regulate proteasome function as suggested by a recent study showing that the UBL domain of DDI2 promotes the ATPase activity of the proteasome [43]. In contrast, our reconstitution experiments showed that the lack of the UBL domain did not impair DDI2 ability to function as an NRF1 activating factor. These observations indicate that aspects of DDI2-mediated proteasome regulatory functions could be separated from its contribution to NRF1 maturation.

We demonstrated that both the RVP protease and HDD domains are essential for DDI2 proteolytic activation of NRF1. This HDD fold may contribute to substrate recruitment. In yeast, the homolog of DDI2, Ddi1, was described to recognize long ubiquitin chains. This process was shown to rely in part on a functional HDD region [44]. The HDD may also be involved in DDI2 activation, the α-helical domain of the HDD region is present in Rad23 and Dsk2, two proteasome shuttle proteins that interact with the proteasome [32]. This region may therefore interact with the proteasome, possibly sensing its overall fitness to regulate effector mechanisms. The crucial role of the HDD in mediating NRF1 maturation is also supported by the fact that, among RVP-containing proteins, DDI1, the only one harboring an HDD, was found to restore NRF1 activation. DDI1 is a paralogue of DDI2; however, while this protein retains function, its expression is undetectable in human tissues. By immunoblotting, we could not detect the presence of this protein in MM, including in cells with DDI2 deficiency. However, we cannot exclude that in specific contexts, DDI1 may compensate for the lack of DDI2.

A functional RVP domain is required for DDI2 proteolytic activity. In this study, we found that in MM, targeting the RVP domain with the anti-HIV drug NFV, blocked basal maturation of NRF1 and partially decreased DDI2 activity upon treatment with BTZ. These observations suggest that NFV could be repositioned in MM to restore sensitivity to BTZ. In support of this hypothesis, phase II clinical trials have provided promising clinical evidence that NFV may resensitize proteasome inhibitor-refractory MM to proteasome inhibition [45–47]. Our data demonstrating that DDI2 can contribute to proteasome inhibition adaptation may explain this response. NFV-mediated DDI2 inhibition likely disrupts a mechanism of resistance in MM. In addition, NFV could exacerbate BTZ effects by blocking the NRF1-dependent proteasome bounce-back response. However, this interpretation should be cautioned by the fact that NFV has additional effects, including modulation of translation mechanisms that also contribute to its antitumoral properties [21, 38, 48].

To clarify this issue, the development of specific DDI2 inhibitors will help determine the beneficial effects of impairing DDI2 in MM. Based on the genetic studies presented in this study, we can speculate that DDI2-specific drugs could restore responses to BTZ in resistant tumors. In addition, as part of the first line of treatment, this strategy could also synergize with BTZ therapies, possibly increasing its activity and thereby decreasing the rates of MM resistance and relapse.

## Supplementary information

Supplementary Figures and Methods

Immunoblots experimental replicates

Original Data File

Reproducibility checklist

## Data Availability

All data supporting the findings of this study are available from the corresponding author on reasonable request.

## References

[1] Palumbo A, Anderson K (2011). Multiple myeloma. N Engl J Med.

[2] Kazandjian D (2016). Multiple myeloma epidemiology and survival: a unique malignancy. Semin Oncol.

[3] Honjo T, Alt FW, Neuberger MS. Molecular biology of B cells. Amsterdam: Elsevier; 2004. xiv, pp. 589.

[4] Hipp MS, Kasturi P, Hartl FU (2019). The proteostasis network and its decline in ageing. Nat Rev Mol Cell Biol.

[5] Collins GA, Goldberg AL (2017). The logic of the 26S proteasome. Cell..

[6] Adams J (2004). The development of proteasome inhibitors as anticancer drugs. Cancer Cell.

[7] Hideshima T, Richardson P, Chauhan D, Palombella VJ, Elliott PJ, Adams J (2001). The proteasome inhibitor PS-341 inhibits growth, induces apoptosis, and overcomes drug resistance in human multiple myeloma cells. Cancer Res.

[8] Okazuka K, Ishida T (2018). Proteasome inhibitors for multiple myeloma. Jpn J Clin Oncol.

[9] Besse A, Besse L, Kraus M, Mendez-Lopez M, Bader J, Xin BT (2019). Proteasome inhibition in multiple myeloma: head-to-head comparison of currently available proteasome inhibitors. Cell Chem Biol.

[10] Niewerth D, Jansen G, Assaraf YG, Zweegman S, Kaspers GJ, Cloos J (2015). Molecular basis of resistance to proteasome inhibitors in hematological malignancies. Drug Resist Updat.

[11] Gonzalez-Santamarta M, Quinet G, Reyes-Garau D, Sola B, Roue G, Rodriguez MS (2020). Resistance to the proteasome inhibitors: lessons from multiple myeloma and mantle cell lymphoma. Adv Exp Med Biol.

[12] Radhakrishnan SK, Lee CS, Young P, Beskow A, Chan JY, Deshaies RJ (2010). Transcription factor Nrf1 mediates the proteasome recovery pathway after proteasome inhibition in mammalian cells. Mol Cell.

[13] Li X, Matilainen O, Jin C, Glover-Cutter KM, Holmberg CI, Blackwell TK (2011). Specific SKN-1/Nrf stress responses to perturbations in translation elongation and proteasome activity. PLoS Genet.

[14] Steffen J, Seeger M, Koch A, Kruger E (2010). Proteasomal degradation is transcriptionally controlled by TCF11 via an ERAD-dependent feedback loop. Mol Cell.

[15] Sha Z, Goldberg AL (2014). Proteasome-mediated processing of Nrf1 is essential for coordinate induction of all proteasome subunits and p97. Curr Biol.

[16] Hagenbuchner J, Ausserlechner MJ (2016). Targeting transcription factors by small compounds—current strategies and future implications. Biochem Pharmacol.

[17] Tomlin FM, Gerling-Driessen UIM, Liu YC, Flynn RA, Vangala JR, Lentz CS (2017). Inhibition of NGLY1 inactivates the transcription factor Nrf1 and potentiates proteasome inhibitor cytotoxicity. ACS Cent Sci.

[18] Lehrbach NJ, Breen PC, Ruvkun G (2019). Protein sequence editing of SKN-1A/Nrf1 by peptide:N-glycanase controls proteasome gene expression. Cell..

[19] Koizumi S, Irie T, Hirayama S, Sakurai Y, Yashiroda H, Naguro I (2016). The aspartyl protease DDI2 activates Nrf1 to compensate for proteasome dysfunction. elife.

[20] Northrop A, Vangala JR, Feygin A, Radhakrishnan SK (2020). Disabling the protease DDI2 attenuates the transcriptional activity of NRF1 and potentiates proteasome inhibitor cytotoxicity. Int J Mol Sci.

[21] De Gassart A, Bujisic B, Zaffalon L, Decosterd LA, Di Micco A, Frera G (2016). An inhibitor of HIV-1 protease modulates constitutive eIF2alpha dephosphorylation to trigger a specific integrated stress response. Proc Natl Acad Sci USA.

[22] Meerbrey KL, Hu G, Kessler JD, Roarty K, Li MZ, Fang JE (2011). The pINDUCER lentiviral toolkit for inducible RNA interference in vitro and in vivo. Proc Natl Acad Sci USA.

[23] Greenstein S, Krett NL, Kurosawa Y, Ma C, Chauhan D, Hideshima T (2003). Characterization of the MM.1 human multiple myeloma (MM) cell lines: a model system to elucidate the characteristics, behavior, and signaling of steroid-sensitive and -resistant MM cells. Exp Hematol.

[24] Bianchi G, Oliva L, Cascio P, Pengo N, Fontana F, Cerruti F (2009). The proteasome load versus capacity balance determines apoptotic sensitivity of multiple myeloma cells to proteasome inhibition. Blood..

[25] Shabaneh TB, Downey SL, Goddard AL, Screen M, Lucas MM, Eastman A (2013). Molecular basis of differential sensitivity of myeloma cells to clinically relevant bolus treatment with bortezomib. PLoS ONE.

[26] Canturk Z, Dikmen M, Artagan O, Ozarda MG, Ozturk N (2016). Cytotoxic effects of resveratrol, rutin and rosmarinic acid on ARH-77 human (multiple myeloma) cell line. Nat Prod Commun.

[27] Drewinko B, Mars W, Minowada J, Burk KH, Trujillo JM (1984). ARH-77, an established human IgG-producing myeloma cell line. I. Morphology, B-cell phenotypic marker profile, and expression of Epstein-Barr virus. Cancer..

[28] Drexler HG, Matsuo Y (2000). Malignant hematopoietic cell lines: in vitro models for the study of multiple myeloma and plasma cell leukemia. Leuk Res.

[29] Chen T, Ho M, Briere J, Moscvin M, Czarnecki PG, Anderson KC (2022). Multiple myeloma cells depend on the DDI2/NRF1-mediated proteasome stress response for survival. Blood Adv.

[30] Shimizu S, Takiguchi T, Fukutoku M, Yoshioka R, Hirose Y, Fukuhara S (1993). Establishment of a CD4-positive plasmacytoma cell line (AMO1). Leukemia..

[31] Gu Y, Wang X, Wang Y, Wang Y, Li J, Yu FX (2020). Nelfinavir inhibits human DDI2 and potentiates cytotoxicity of proteasome inhibitors. Cell Signal.

[32] Siva M, Svoboda M, Veverka V, Trempe JF, Hofmann K, Kozisek M (2016). Human DNA-damage-inducible 2 protein is structurally and functionally distinct from its yeast ortholog. Sci Rep.

[33] Bernard D, Mehul B, Thomas-Collignon A, Delattre C, Donovan M, Schmidt R (2005). Identification and characterization of a novel retroviral-like aspartic protease specifically expressed in human epidermis. J Invest Dermatol.

[34] Matsui T, Kinoshita-Ida Y, Hayashi-Kisumi F, Hata M, Matsubara K, Chiba M (2006). Mouse homologue of skin-specific retroviral-like aspartic protease involved in wrinkle formation. J Biol Chem.

[35] Kawabata S, Gills JJ, Mercado-Matos JR, Lopiccolo J, Wilson W, Hollander MC (2012). Synergistic effects of nelfinavir and bortezomib on proteotoxic death of NSCLC and multiple myeloma cells. Cell Death Dis.

[36] Kraus M, Bader J, Overkleeft H, Driessen C (2013). Nelfinavir augments proteasome inhibition by bortezomib in myeloma cells and overcomes bortezomib and carfilzomib resistance. Blood Cancer J.

[37] Gills JJ, Lopiccolo J, Tsurutani J, Shoemaker RH, Best CJ, Abu-Asab MS (2007). Nelfinavir, A lead HIV protease inhibitor, is a broad-spectrum, anticancer agent that induces endoplasmic reticulum stress, autophagy, and apoptosis in vitro and in vivo. Clin Cancer Res.

[38] De Gassart A, Demaria O, Panes R, Zaffalon L, Ryazanov AG, Gilliet M (2016). Pharmacological eEF2K activation promotes cell death and inhibits cancer progression. EMBO Rep.

[39] Fassmannova D, Sedlak F, Sedlacek J, Spicka I, Grantz Saskova K (2020). Nelfinavir inhibits the TCF11/Nrf1-mediated proteasome recovery pathway in multiple myeloma. Cancers (Basel).

[40] Guan M, Su L, Yuan YC, Li H, Chow WA (2015). Nelfinavir and nelfinavir analogs block site-2 protease cleavage to inhibit castration-resistant prostate cancer. Sci Rep.

[41] Ge M, Qiao Z, Kong Y, Liang H, Sun Y, Lu H (2021). Modulating proteasome inhibitor tolerance in multiple myeloma: an alternative strategy to reverse inevitable resistance. Br J Cancer.

[42] Netea MG, Dominguez-Andres J, Barreiro LB, Chavakis T, Divangahi M, Fuchs E (2020). Defining trained immunity and its role in health and disease. Nat Rev Immunol.

[43] Collins GA, Goldberg AL (2020). Proteins containing ubiquitin-like (Ubl) domains not only bind to 26S proteasomes but also induce their activation. Proc Natl Acad Sci USA.

[44] Yip MCJ, Bodnar NO, Rapoport TA (2020). Ddi1 is a ubiquitin-dependent protease. Proc Natl Acad Sci USA.

[45] Driessen C, Muller R, Novak U, Cantoni N, Betticher D, Mach N (2018). Promising activity of nelfinavir-bortezomib-dexamethasone in proteasome inhibitor-refractory multiple myeloma. Blood..

[46] Erath A, Patel DA, Hosack EA, Patanella JE, Ibach DM, Kassim AA (2020). Overcoming proteasome inhibitor-refractory multiple myeloma with elotuzumab, bortezomib, nelfinavir, and dexamethasone. World J Oncol.

[47] Hitz F, Kraus M, Pabst T, Hess D, Besse L, Silzle T (2019). Nelfinavir and lenalidomide/dexamethasone in patients with lenalidomide-refractory multiple myeloma. A phase I/II Trial (SAKK 39/10). Blood Cancer J.

[48] Besse L, Besse A, Stolze SC, Sobh A, Zaal EA, van der Ham AJ (2021). Treatment with HIV-protease inhibitor nelfinavir identifies membrane lipid composition and fluidity as a therapeutic target in advanced multiple myeloma. Cancer Res.

