# Supplementary material for: The aspartyl protease DDI2 drives adaptation to proteasome inhibition in multiple myeloma
Source: Cell Death Dis. 2022 May 19;13(5):475. doi: 10.1038/s41419-022-04925-3 (PMC9120136; doi:10.1038/s41419-022-04925-3)
Supplement: Supplementary file 1 — Supplementary Figures and Methods [file 41419_2022_4925_MOESM1_ESM.docx]

**Supplementary information for:**

**The aspartyl protease DDI2 drives adaptation to proteasome inhibition in multiple myeloma**

Mélanie Op^1^, Sérgio T. Ribeiro^1^, Claire Chavaria^1^, Aude de Gassart^1,2^, Léa Zaffalon^1^, and Fabio Martinon^1*^

^1^ Dept. of Biochemistry, University of Lausanne, 155 Ch. des Boveresses, Epalinges 1066, Switzerland

^2^ present address: ImCheck Therapeutics, Marseille, 13009, France

**Supplementary figures S1; S2; S3**

**Supplementary Methods**

**
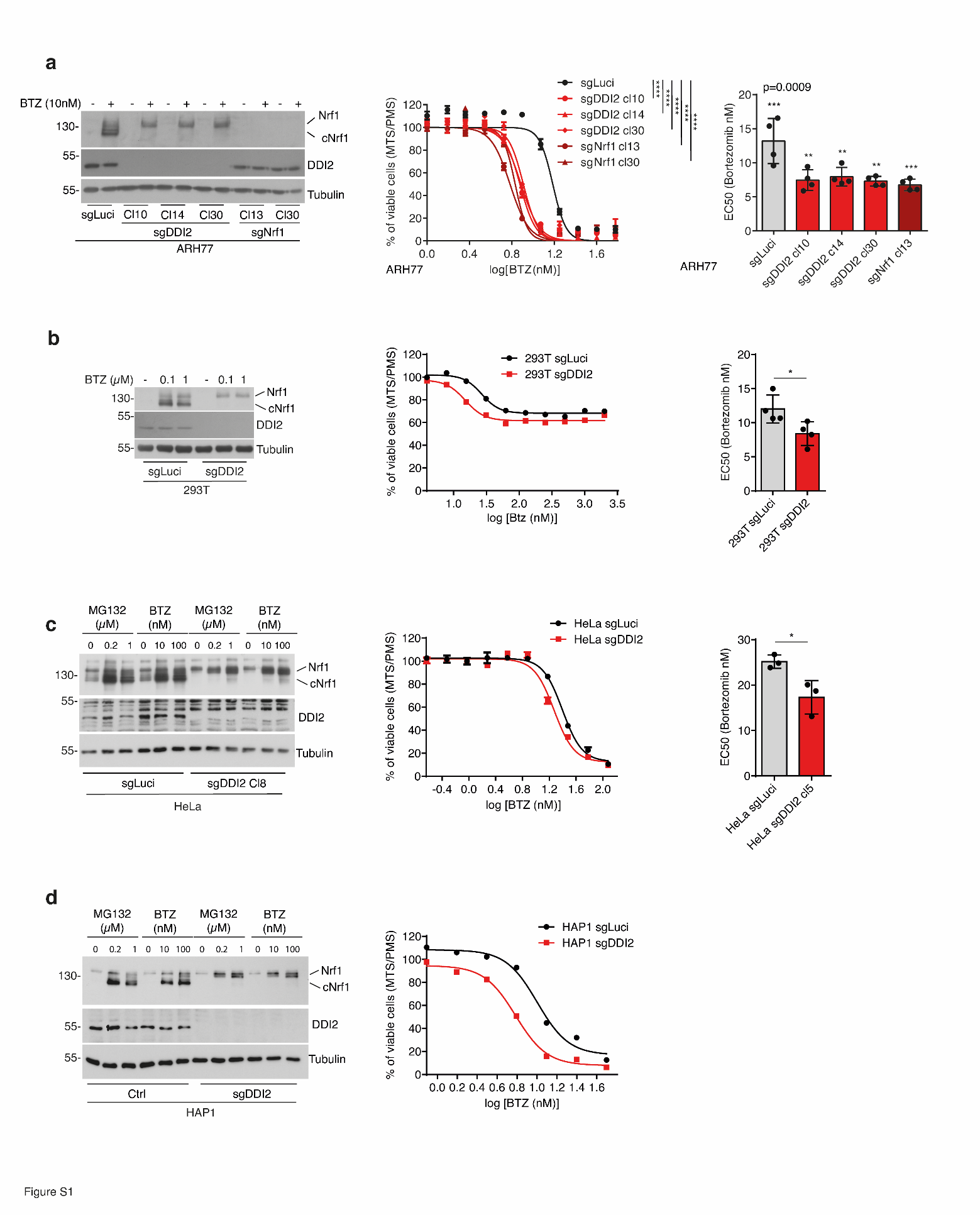
**

**Figure S1: DDI2 deficiency affects Bortezomib sensitivity of common cell lines**

**a** ARH77 population expressing control luciferase (Luci) sgRNA or representative clonal DDI2 deficient populations and representative clonal NRF1 deficient populations were treated with BTZ or vehicle as indicated and analyzed by immunoblotting for expression of DDI2 and NRF1, cNRF1, indicates cleaved NRF1. Sensitivity to BTZ of control cells and representative DDI2 or NRF1 deficient clones was analyzed by viability assay. **b-d** 293T, HeLa, and HAP1 populations expressing control luciferase (Luci) sgRNA or representative DDI2 deficient populations were treated with BTZ or vehicle as indicated and analyzed by immunoblotting for expression of DDI2 and NRF1. Sensitivity to BTZ was analyzed by viability assay. Curve graphs are from one representative experiment of three performed in triplicate. P-values were calculated using two-way ANOVA between control cells and KO cells. Dots graph represents the EC50 (half-maximal effective concentration) of the dose responses; data are from at least three independent experiments performed in triplicate. P-values were calculated using one-way ANOVA followed by Dunnett’s multiple comparison tests.


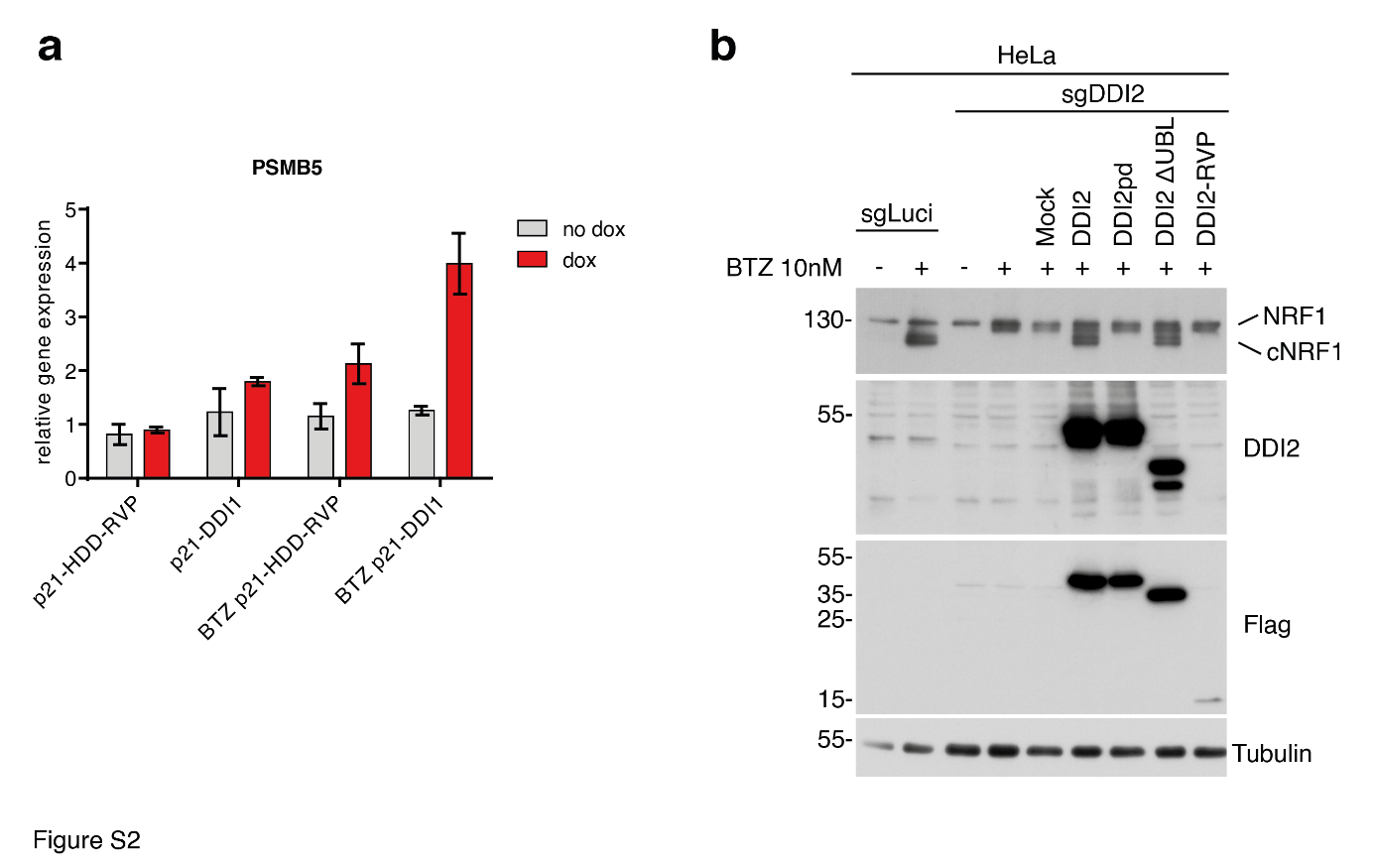


**Figure S2: DDI2 reconstitution in DDI2-deficient HeLa cells**

**a** Indicated ARH77 cells were primed or not with doxycycline and treated 24 h with 10 nM BTZ and analyzed for PSMB5 mRNA expression by real-time PCR. Normalization was done relative to HPRT levels (representative of 2). **b** The protein expression of NRF1, DDI2, and FLAG was analyzed by Western Blot in HeLa cells transfected with some of the FLAG-DDI2 constructs. Doxycycline is used to induce their expressions. Tubulin is used as a loading control.


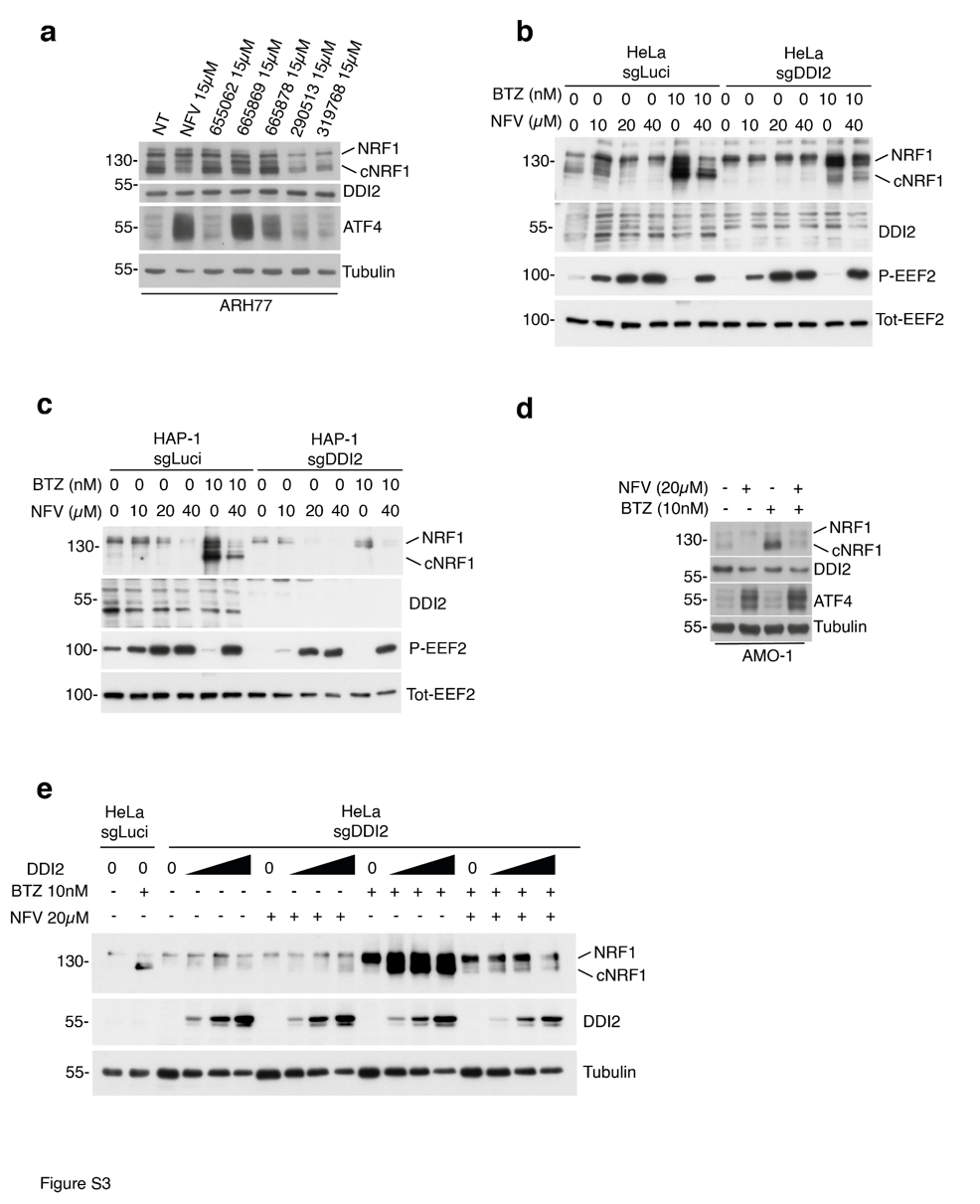


**Figure S3: Effects of nelfinavir analogues effects on NRF1 maturation**

**a** ARH77 were treated with different analogues of nelfinavir (NFV) identified in the NCI Open Chemical Repository Collection. The protein expression of DDI2, NRF1, and ATF4 after treatment was assessed by Immunoblotting. cNRF1 indicates cleaved NRF1. Tubulin is used as a loading control. **b-c** Indicated cells were treated with increased doses of NFV, in combination with BTZ. The protein expression of DDI2, NRF1, P-EEF2 after treatment was assessed by Immunoblotting. cNRF1 indicates cleaved NRF1. Tot-EEF2 was used as a loading control. **d** Parental AMO-1 cells were treated with BTZ and NFV. The protein expression of DDI2, NRF1, and ATF4 after treatment was assessed by Immunoblotting. cNRF1 indicates cleaved NRF1. Tubulin is used as a loading control. **c** A representative HeLa clone expressing DDI2 sgRNA and deficient for DDI2 expression was reconstituted with an inducible FLAG-DDI2 construct (backbone PINDUCER21) and treated with BTZ and increased doses of doxycycline (Dox) as indicated. The expression of DDI2 and NRF1 was monitored by immunoblotting.

**Materials and methods**

**Antibodies and materials:**

Bortezomib and Carfilzomib were from LC-Laboratories. Nelfinavir Mesylate (CAS 159989-65-8) was from Axon Medchem. 1,4-Dithiothreitol (DTT) and Doxycycline were from AppliChem. The anti-NRF1 (8052), anti-PSMB5 (12919) were purchased from Cell Signaling. The anti-FLAG (F425) was purchased from Sigma. The anti-ATF4 (sc-200) was purchased from Santa Cruz. The anti-Tubulin (AG-27B-0005-C100) was purchased from Adipogen. The anti-DDI2 antibody was purified in our lab from human DDI2 immunized-rabbit serum using HiTrap NHS activated HP columns (GE Healthcare).

**Western blotting**

Cells were directly lysed in sodium dodecyl sulfate (SDS) loading buffer (10% glycerol, 2% SDS, 50 mM Tris-HCl pH 6.8, 12.5 mM EDTA, 0.02% Bromophenol Blue) containing 80-120 mM of dithiothreitol (DTT). Extracts were separated by SDS-PAGE and transferred to nitrocellulose blotting membranes (Amersham).

**Proteasome activity assay**

Between 3 and 5.10^6^ cells were lysed in 120µl of Proteasome lysis Buffer (50 mM HEPES pH 7.8, 10mM NaCl, 1.5mM MgCl_2_, 1mM EDTA, 1mM EGTA, 250mM sucrose and 5mM DTT in PBS without Ca^2+^ or Mg^2+^). The cell lysates were sonicated 3 sec using microtip output set on ~3, then they were centrifuged at 16000 RCF for 10min at 4°C. The supernatants were transferred in new tubes.

For the assay, the Proteasome lysis Buffer is complemented with ATP 2 mM. The proteasome substrates used for the assay are Suc-LLVY-AMC chymotrypsin-like activity substrate from Enzo (BML-P802-0005), Z-ARR-AMC trypsin-like activity substrate from Calbiochem (CAS 90468-18-1), and Z-LLE-AMC caspase-like activity substrate from Adipogen (CAS 348086-66-8). The AMC positive control comes from Biovision (#K245-100-4). Each well is filled with 30-50µg of total proteins and one of the proteasome substrates concentrated at 100 µM. During 60 min, the A_360_ex/A_360_em is measured on a fluorescent plate reader at 37°C.

**Chromatin immunoprecipitation:**

This experiment was performed as previously described. (22). Briefly, for each ChIP reaction cells were harvested and fixed in 1% formaldehyde. Reactions were stopped in 0.18 M glycine. The cells were lysed in 0.5% NP-40 buffer (10 mM Tris pH 8, 1 mM EDTA, 0.5% NP-40) which was followed by the second round of lysis in 1% Triton X-100 (10 mM Tris pH 8, 1 mM EDTA, 1% Triton X-100, 0.5% Na-DOC, 0.5% Sarcosyl, 0.5M NaCl). Lysis buffers were supplemented with Protease’s inhibitors cocktail (Roche). Lysates were sonicated using Diagenode's Bioruptor® Sonicator. For each ChIP reaction, 30 μg of chromatin was pre-cleared by co-incubation with Protein G sepharose beads (4 Fast Flow; GE Health Care Life Sciences), BSA, and Salmon Sperm DNA (UltraPureTM Salmon Sperm DNA Solution; ThermoFisher). Next, a fraction (10%) of pre-cleared chromatin was kept as input and the remaining part of the chromatin was incubated overnight with antibodies. The next day, immunoprecipitation was done using Protein G Sepharose beads. After washes beads were resuspended in elution buffer (111mM Tris pH 8, 1.11% SDS), heated, and treated overnight with Proteinase K. Following day DNA was extracted by phenol-chloroform-isoamyl alcohol (25:24:1). Real-time PCR was performed using Kapa SYBR Fast qPCR kit. Primer sequences used in ChIP experiments are shown in Table 1.

**Statistical analysis:**

Data from one representative independent experiment is shown. All experiments were performed two or three times, except some adaptation experiments of the MM.1S. Statistical significances were determined using Graph Pad Prism version 6 as described in the figure legends. The error bars are the standard deviation of the sample. Significant differences were considered as follow *P ≤ .05, **P ≤ .01, ***P ≤ .001, or **** P ≤ .0001.

Table 1: List of primers used in this study:

| **purpose** | **Genes** | **Forward primer (5’-3’)** | **Reverse (5’-3)** |
| --- | --- | --- | --- |
| CRISPR | ***hDDI2*** | CACCGGCTCGAAGTCGGCGTCGAC | AAACGGTCGACGCCGACTTCGAGCC |
| CRISPR | ***hNRF1*** | CACCGCTTTCTCGCACCCCGTTGTC | AAACGACAACGGGGTGCGAGAAAGC |
| CRISPR | **luciferase** | CACCGCTTCGAAATGTCCGTTCGGT | AAACACCGAACGGACATTTCGAAGC |
| RT-PCR | ***hGAPDH*** | CGCTCTCTGCTCCTCCTGTT | CCATGGTGTCTGAGCGATGT |
| RT-PCR | ***hSRPR*** | GTCCTGAGAACGGAGTAGAACT | ACCCCTCCCATGCTTCTGAAT |
| RT-PCR | ***hPSMB5*** | AGGAACGCATCTCTGTAGCAG | AGGGCCTCTCTTATCCCAGC |
| RT-PCR | ***hPSMB6*** | CTGATGGCGGGAATCATC | CCAATGGCAAAGGACTGC |
| RT-PCR | ***hPSMD11*** | ATGCAGGGAGGCAGACAG | GGAGCTCTGCCCGGTAAT |
| RT-PCR | ***hPSMD14*** | CCGTGCTGGAGTTCCAAT | TGCCTCCACACTGACACC |
| CHIP | ***hGAPDH*** | TACTAGCGGTTTTACGGGCG | TCGAACAGGAGGAGCAGAGAGCGA |
| CHIP | ***hGata1*** | GCCTCAACTGTGTGTCCCAC | GAAGGTACTGGAAAAGTCAG |
| CHIP | ***PSMB5*** | GGACTCACCGCTAAGGGTTC | CGTCCATGTTGCGTAAGGGA |
| CHIP | ***PSMB6*** | TTCTTTTCCCTTCTGCCGTC | ACTGTCGTAAAGCGCTCTGTC |
| CHIP | ***PSMD11*** | CGGTGTGAGAGCGGTAAGAT | CCGATGGAGTGGAGGATGTC |
| CHIP | ***PSMD14*** | GCTGCTGTTGCCTCTGTCTT | GCCTGCCTTCTGGGTCTTAC |
