# Supplementary material for: The aspartyl protease DDI2 drives adaptation to proteasome inhibition in multiple myeloma
Source: Cell Death Dis. 2022 May 19;13(5):475. doi: 10.1038/s41419-022-04925-3 (PMC9120136; doi:10.1038/s41419-022-04925-3)
Supplement: Supplementary file 2 — Immunoblots experimental replicates [file 41419_2022_4925_MOESM2_ESM.pdf]

Replicas Figure 1b

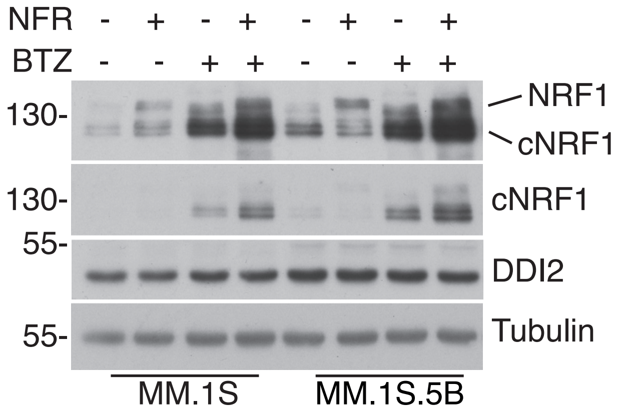

Replica 2

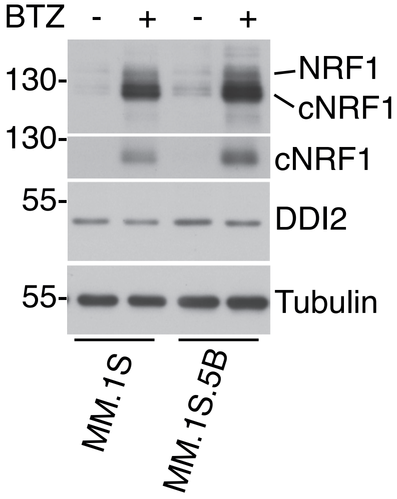

Replica 3

Replicas Figure 1d

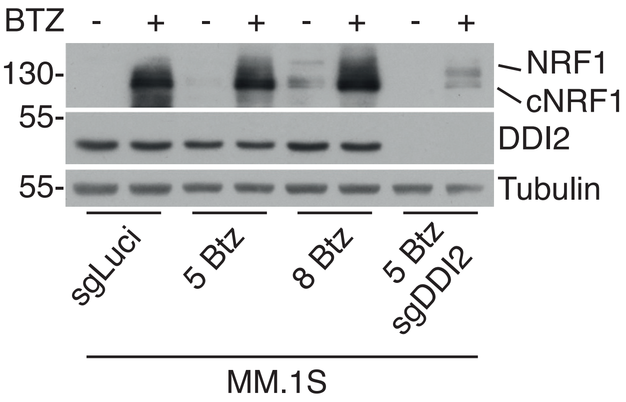

Replica 2

Replicas Figure 2a

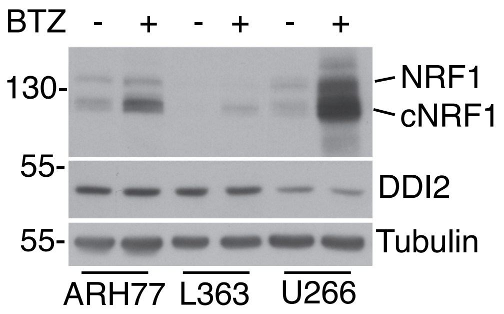

Replica 2
left panel

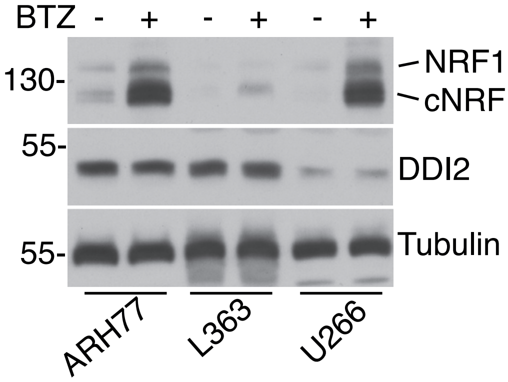

Replica 3
left panel

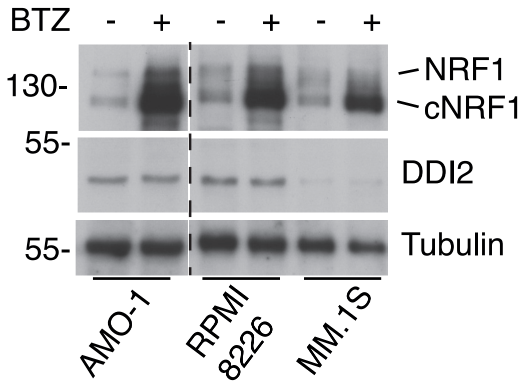

Replica 2
right panel

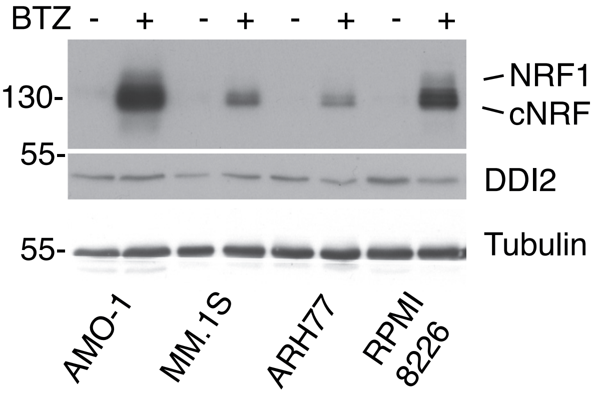

Replica 3
right panel

Replicas Figure 2c

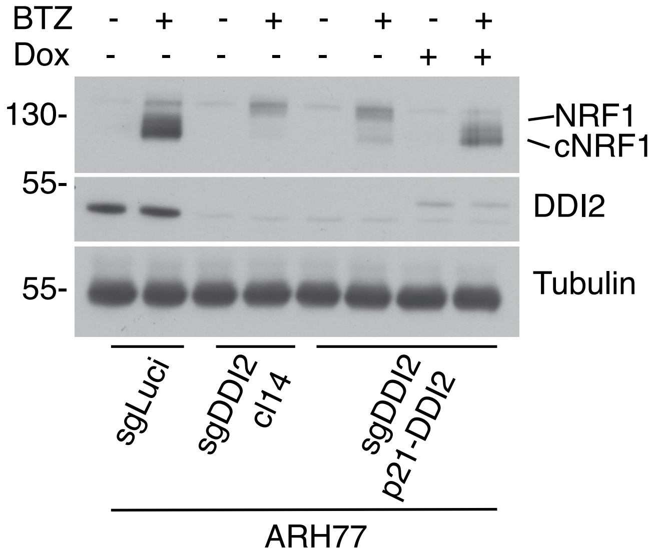

Replica 2

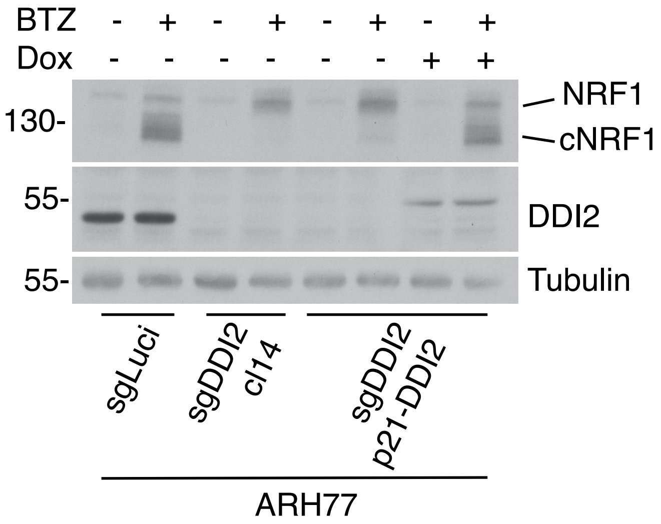

Replica 3

Replicas Figure 2e

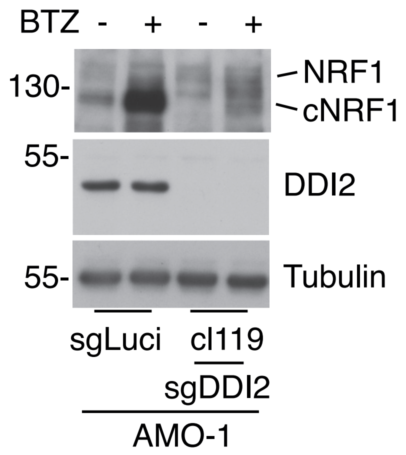

Replica 2

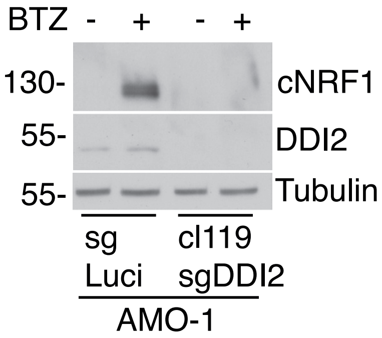

Replica 3

Replicas Figure 2g

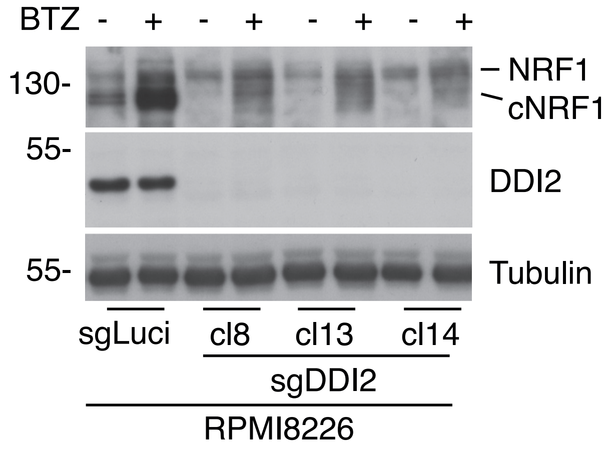

Replica 2

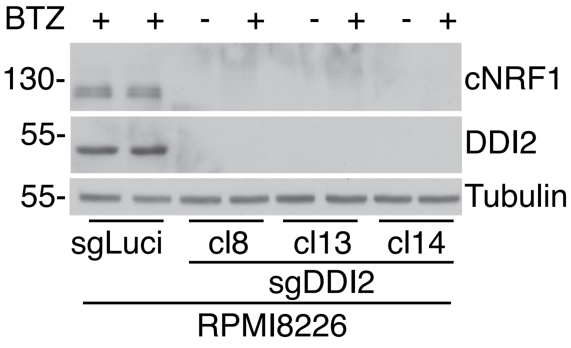

Replica 3

Replica 2 Figure 2i

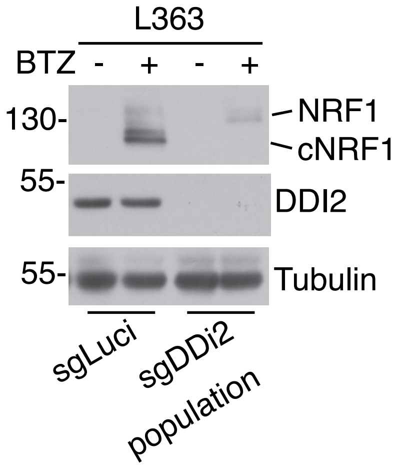

Replicas Figure 4b

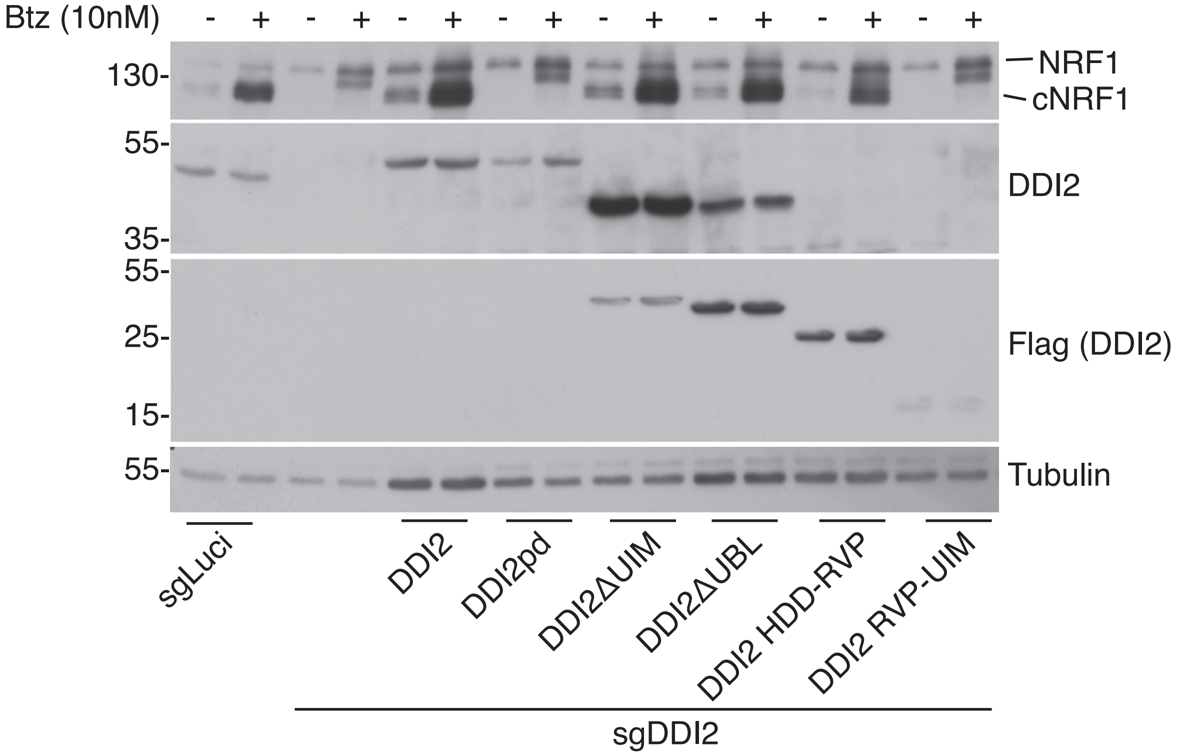

Replica 2

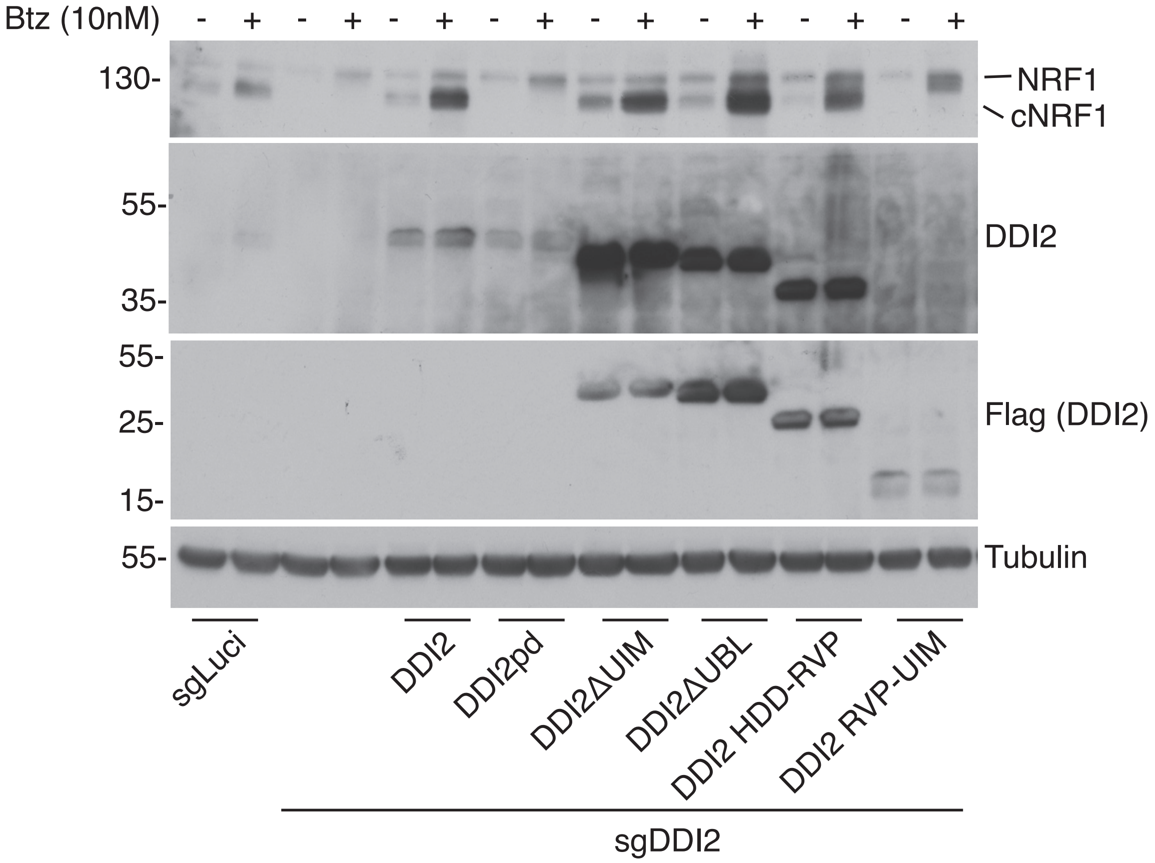

Replica 3

Replica 2 Figure 4d

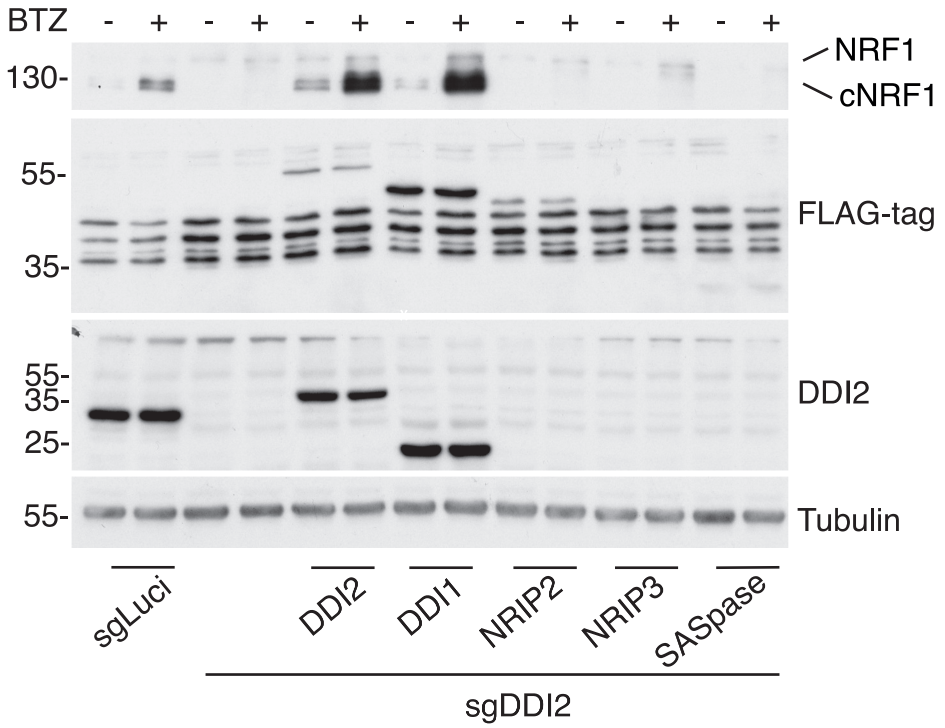

Replicas Figure 5a

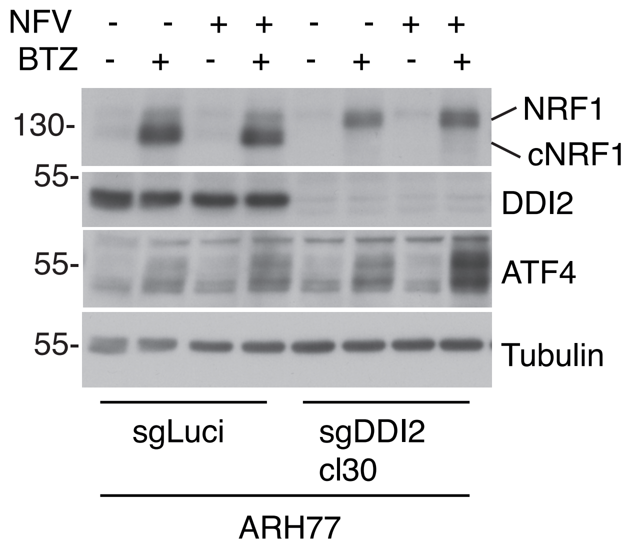

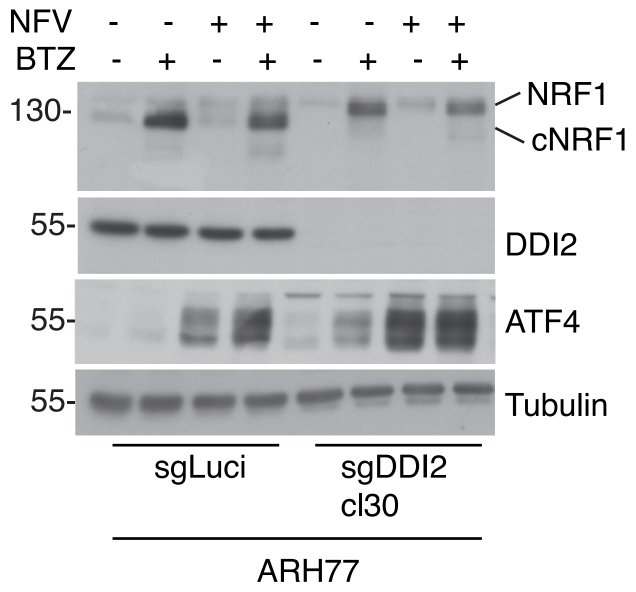

Replica 2

Replica 3
