# Supplementary material for: The aspartyl protease DDI2 drives adaptation to proteasome inhibition in multiple myeloma
Source: Cell Death Dis. 2022 May 19;13(5):475. doi: 10.1038/s41419-022-04925-3 (PMC9120136; doi:10.1038/s41419-022-04925-3)
Supplement: Supplementary file 3 — Original Data File [file 41419_2022_4925_MOESM3_ESM.pdf]

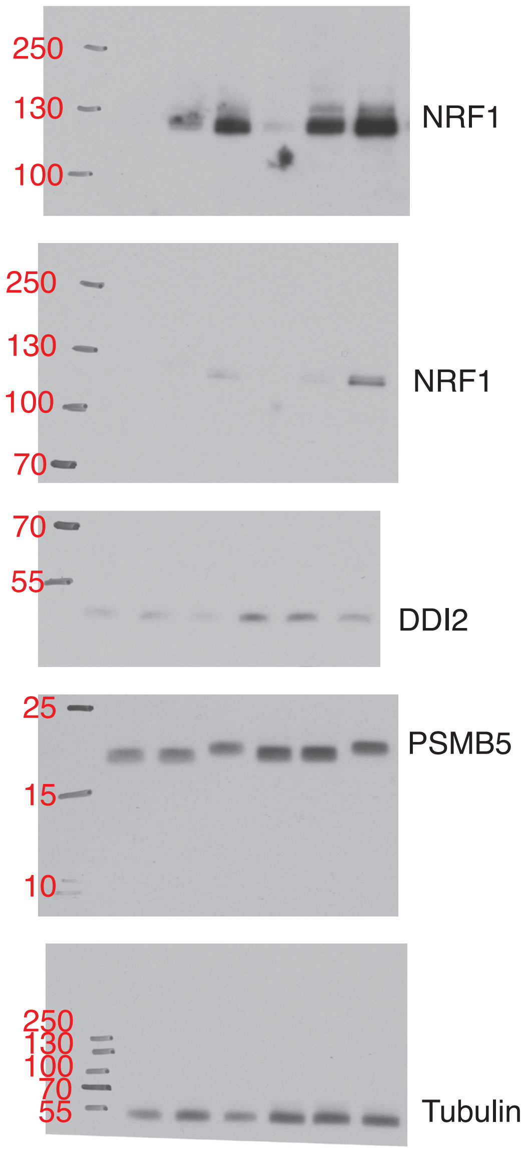

Figure 1b Replica 1

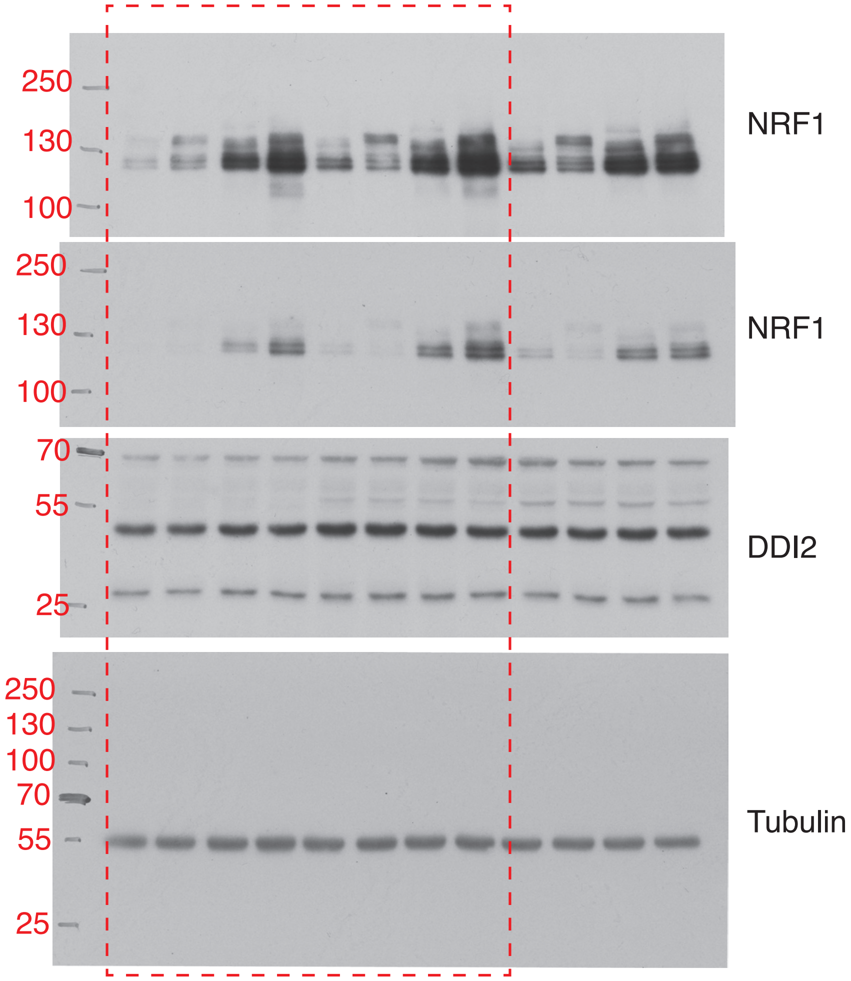

Figure 1b Replica 2

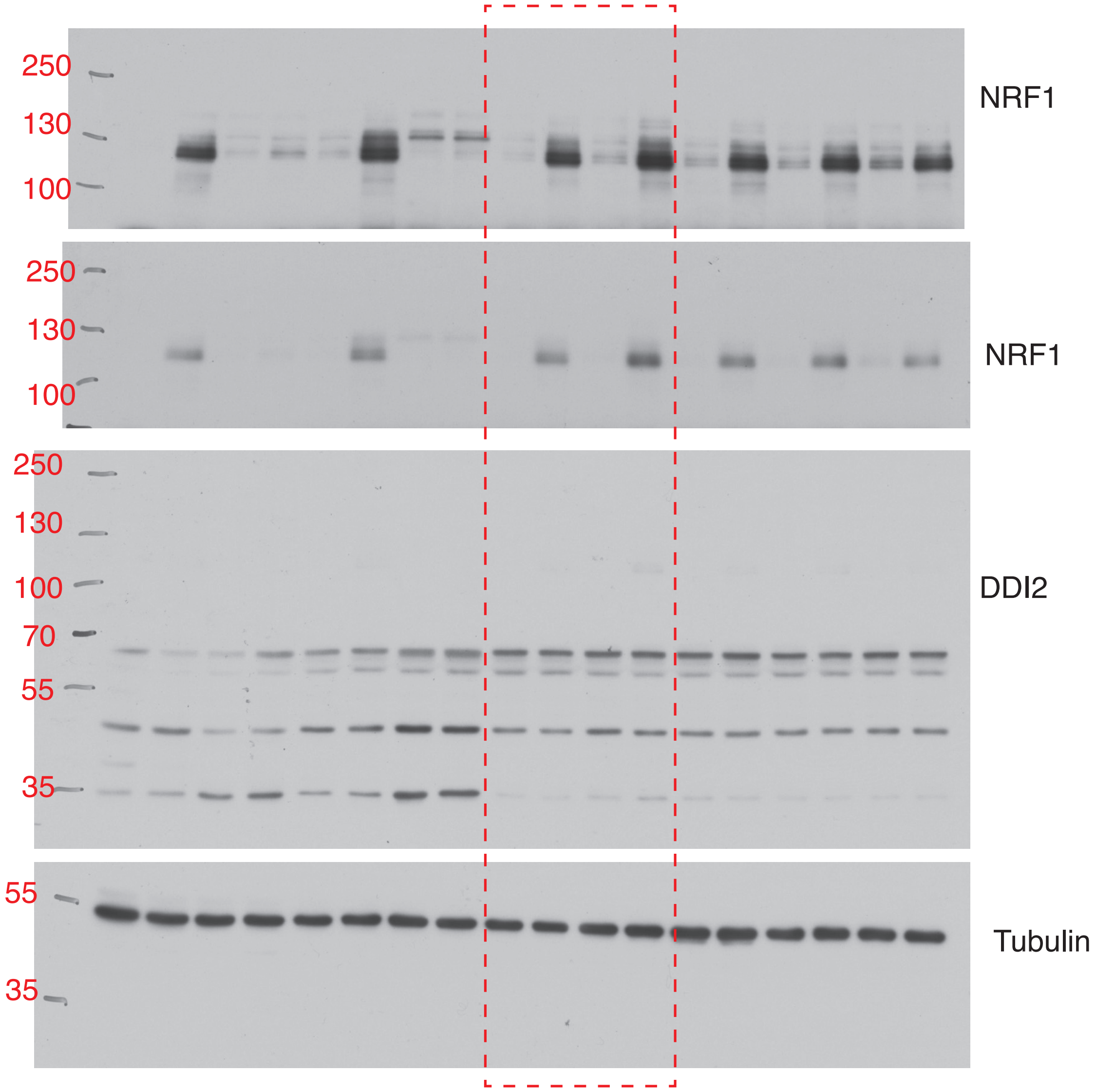

Figure 1b Replica 3

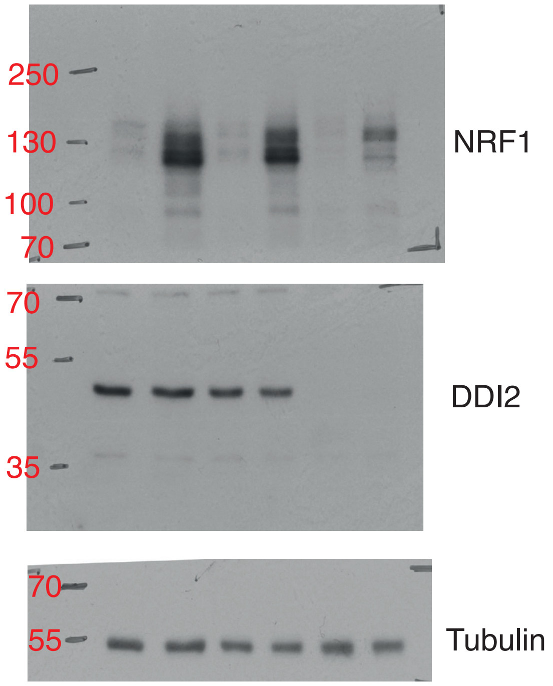

Figure 1d Replica 1

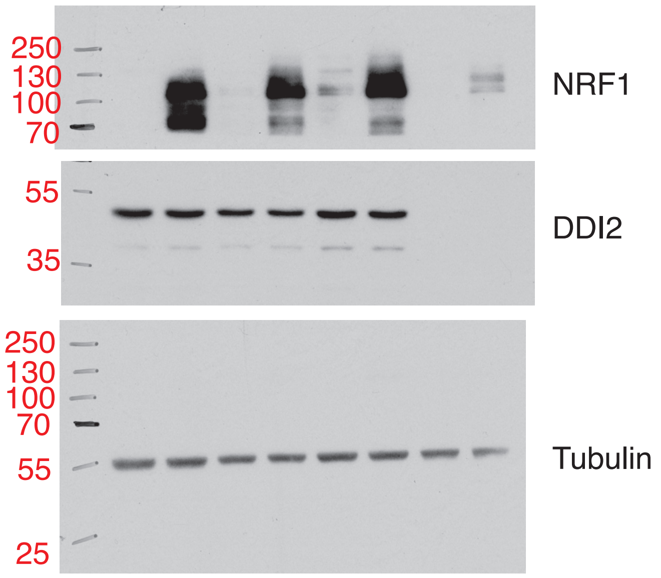

Figure 1d Replica 2

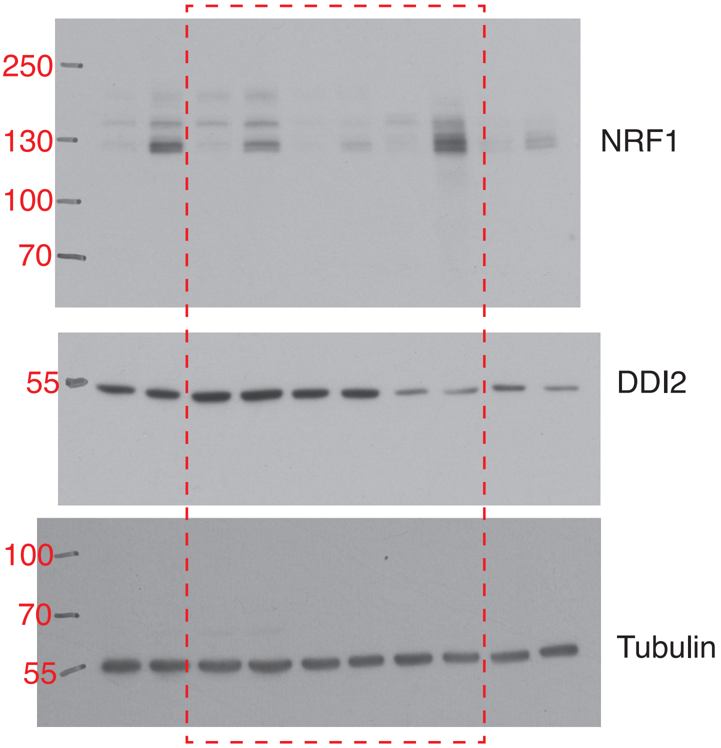

Figure 2a left panel Replica 1

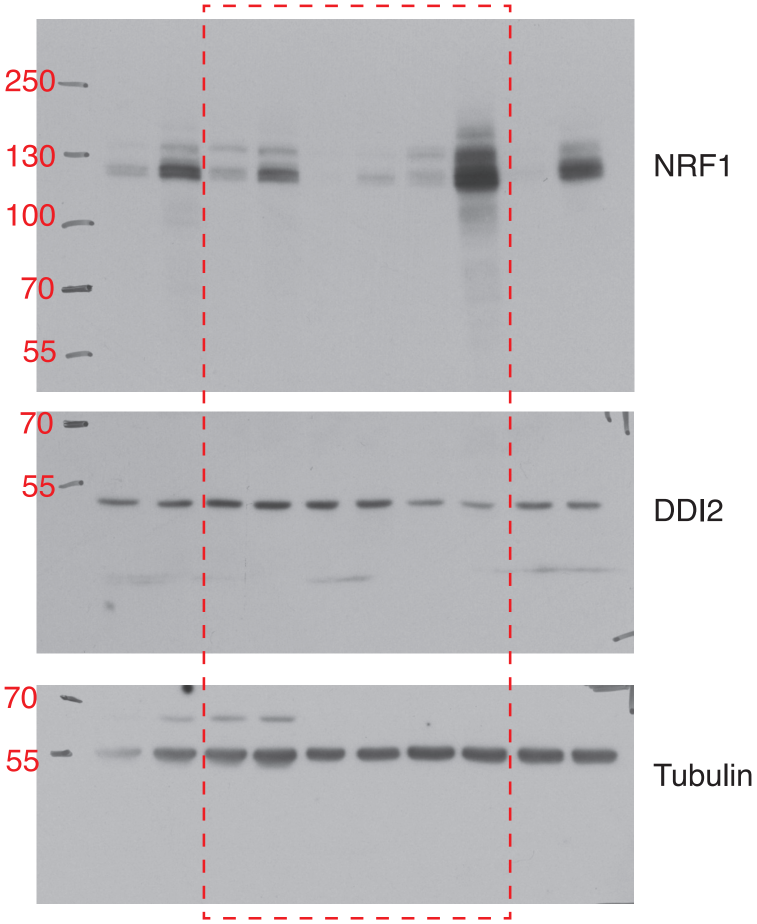

Figure 2a left panel Replica 2

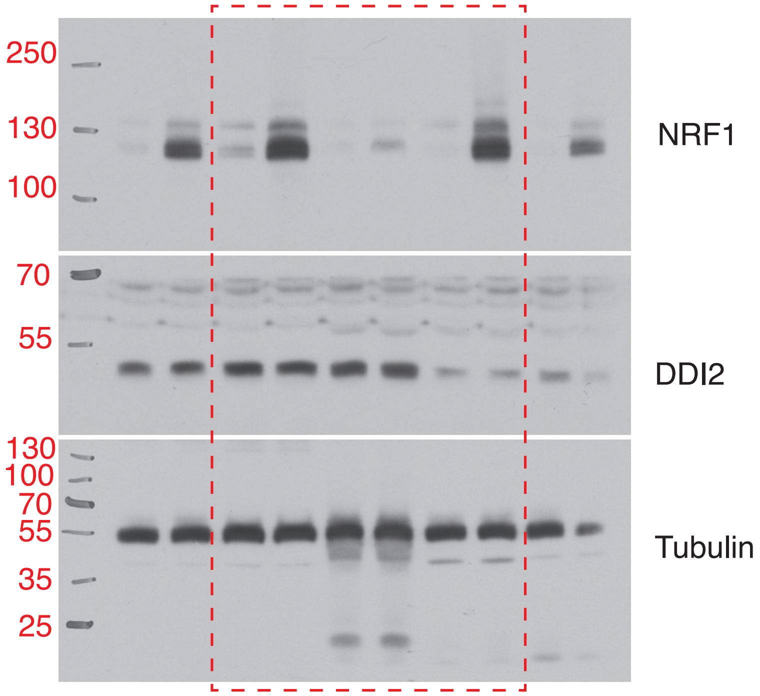

Figure 2a left panel Replica 3

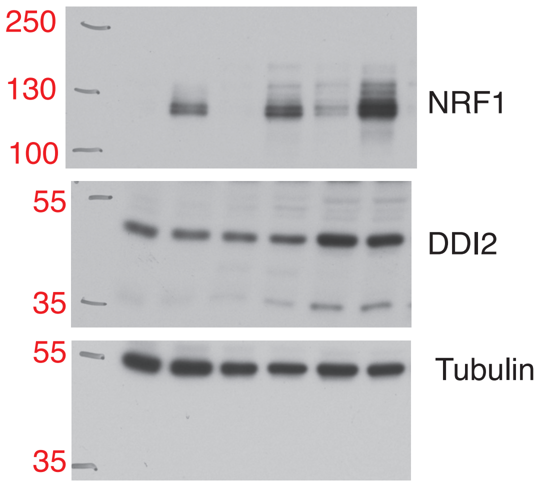

Figure 2a right panel Replica 1

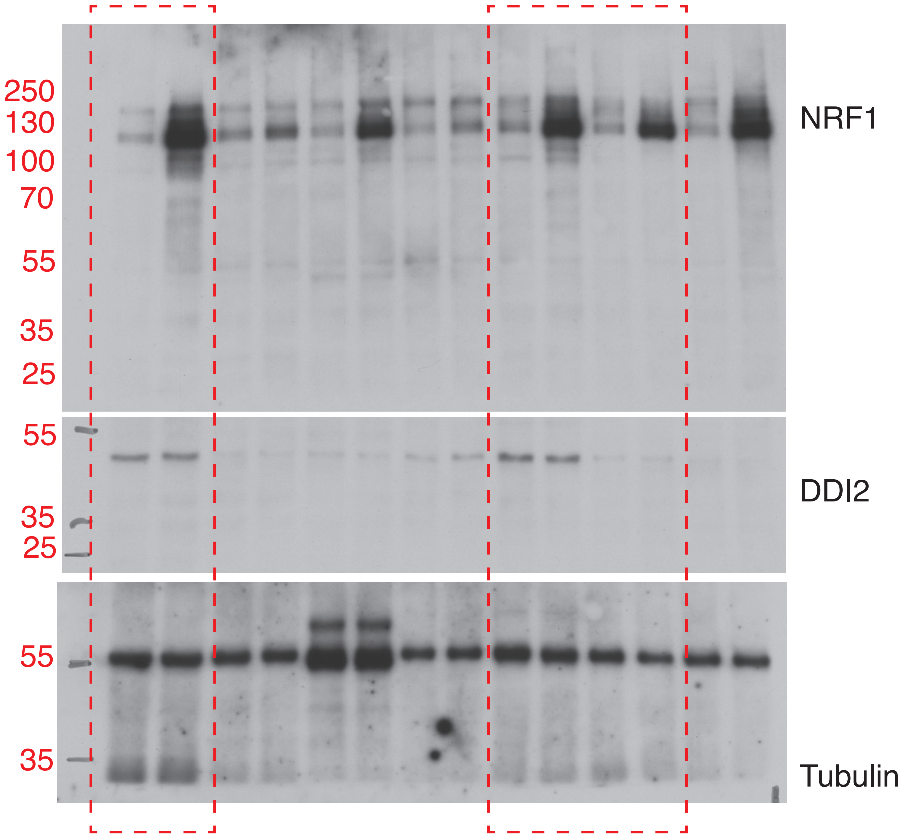

Figure 2a right panel Replica 2

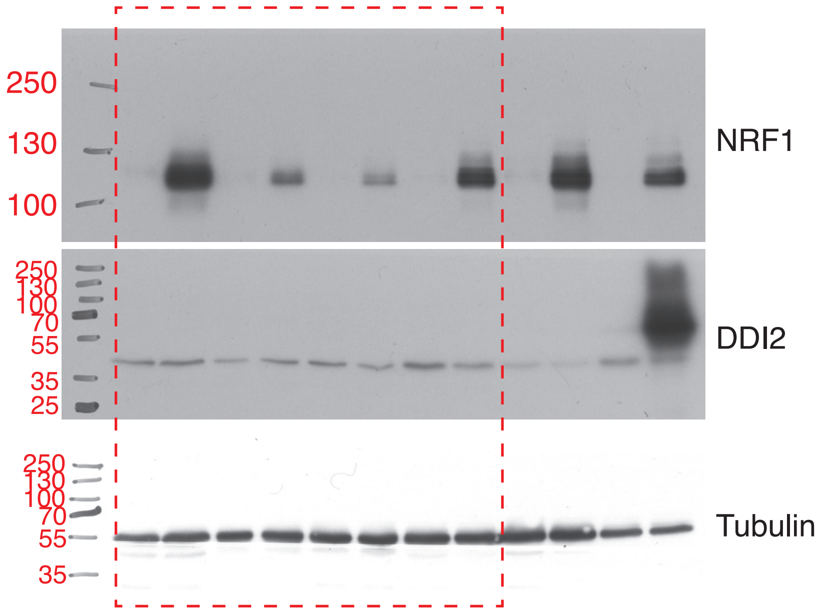

Figure 2a right panel Replica 3

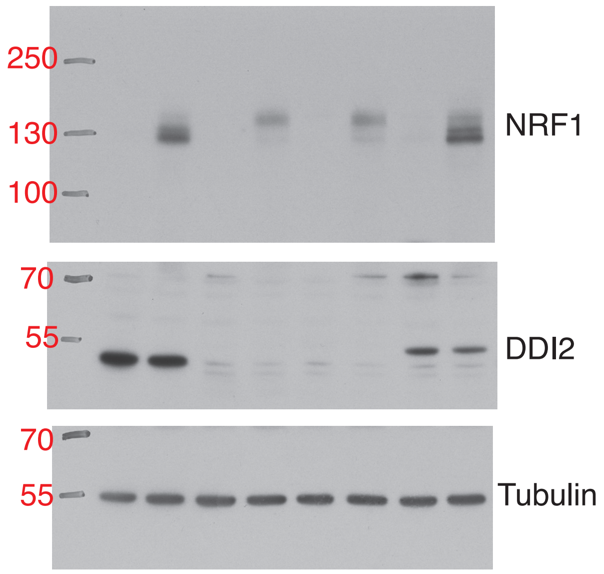

Figure 2c Replica 1

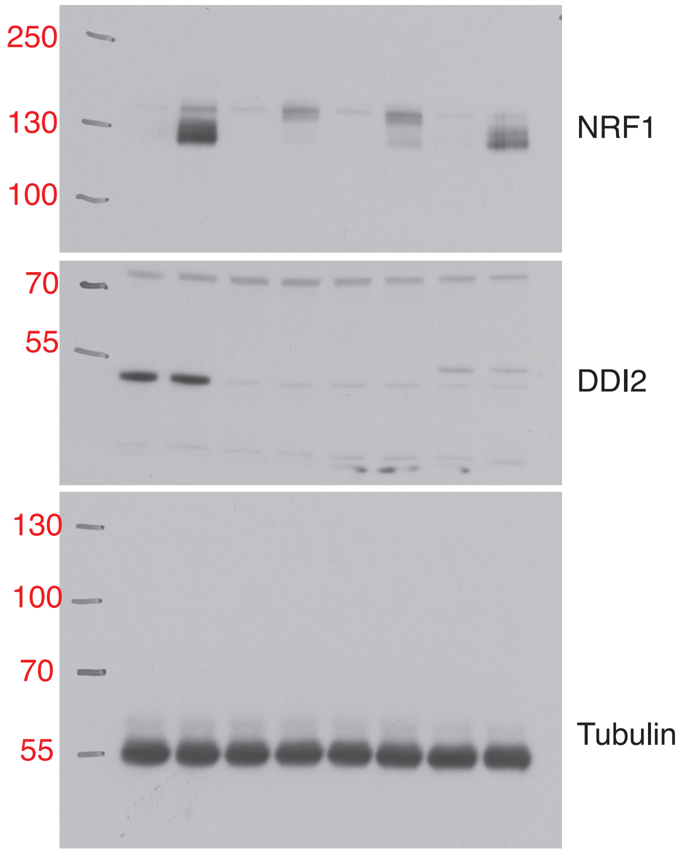

Figure 2c Replica 2

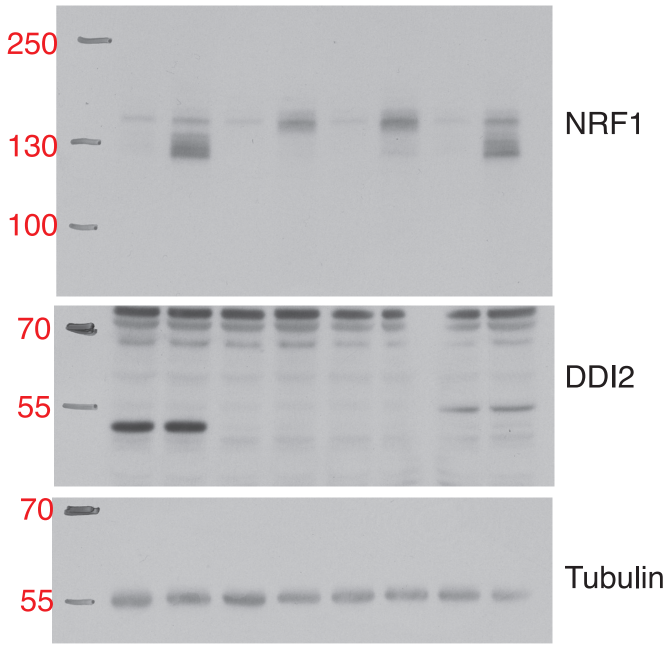

Figure 2c Replica 3

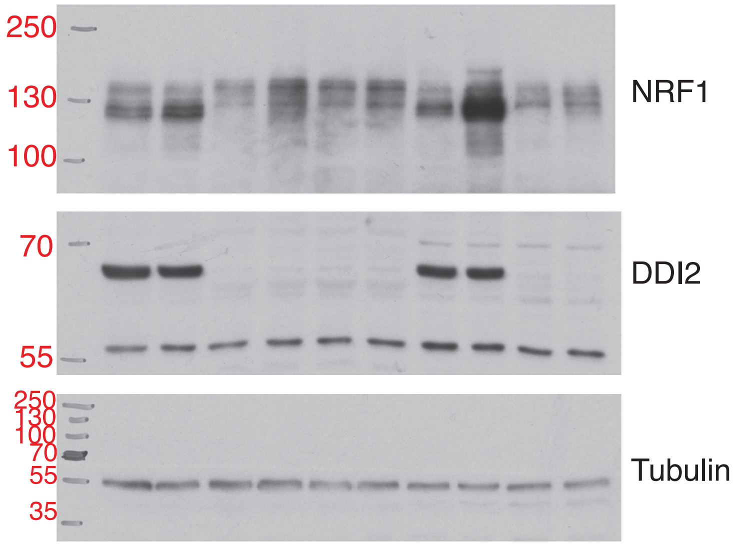

Figure 2e and 2g Replica 1

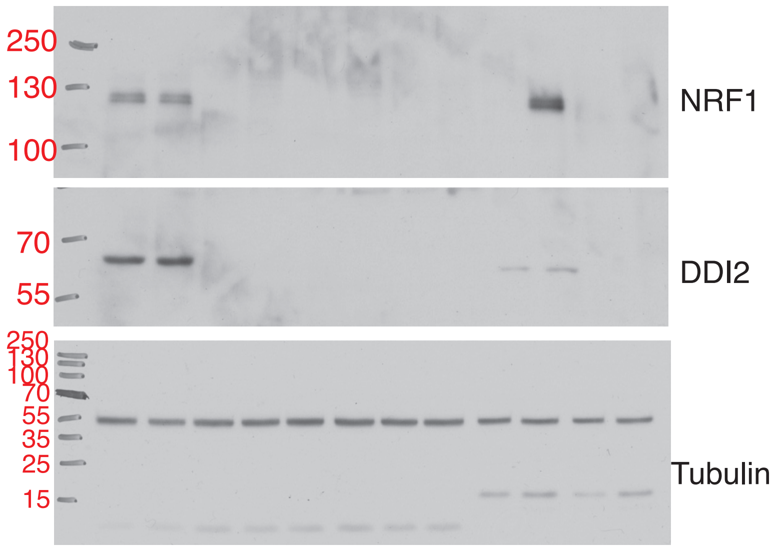

Figure 2e and 2g Replica 3

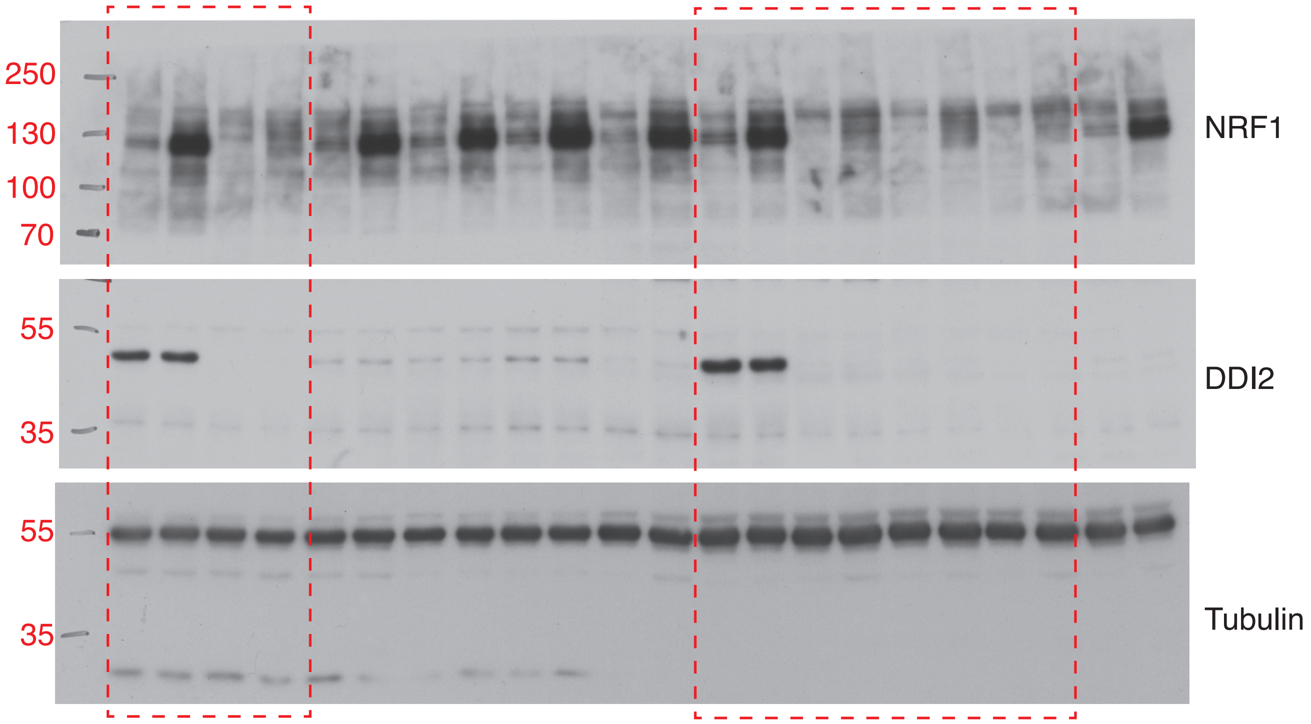

Figure 2e and 2g Replica 2

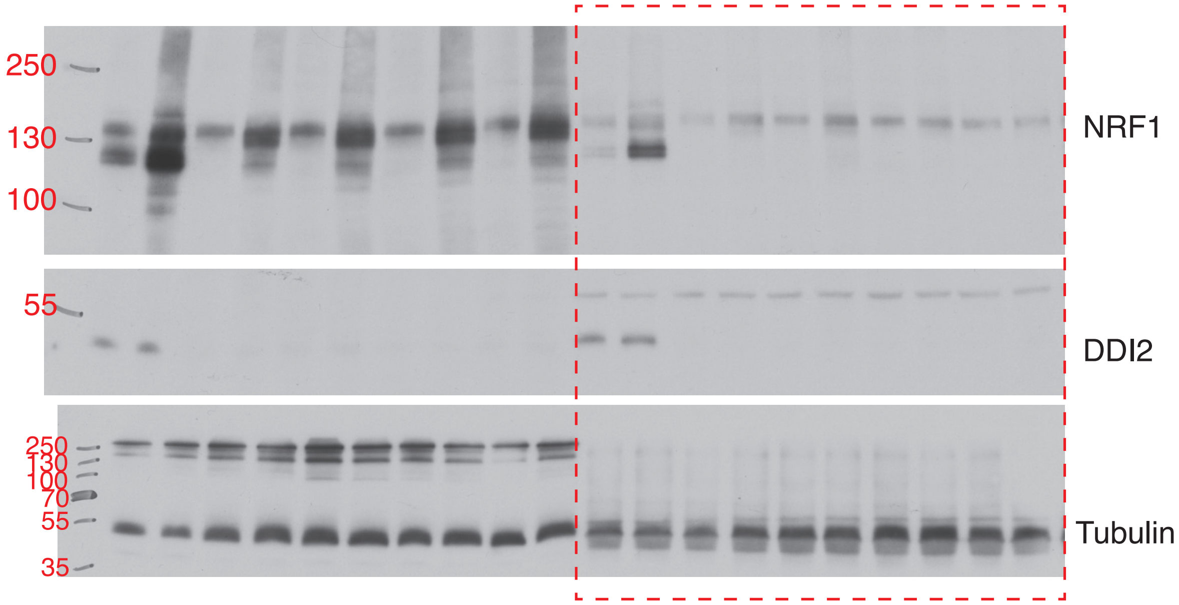

Figure 2i Replica 1

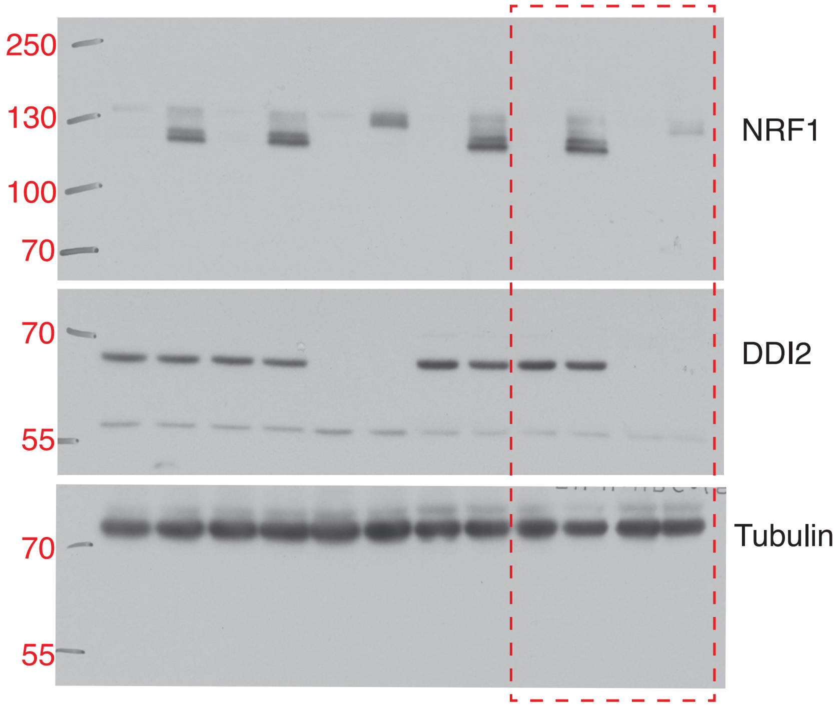

Figure 2i Replica 2

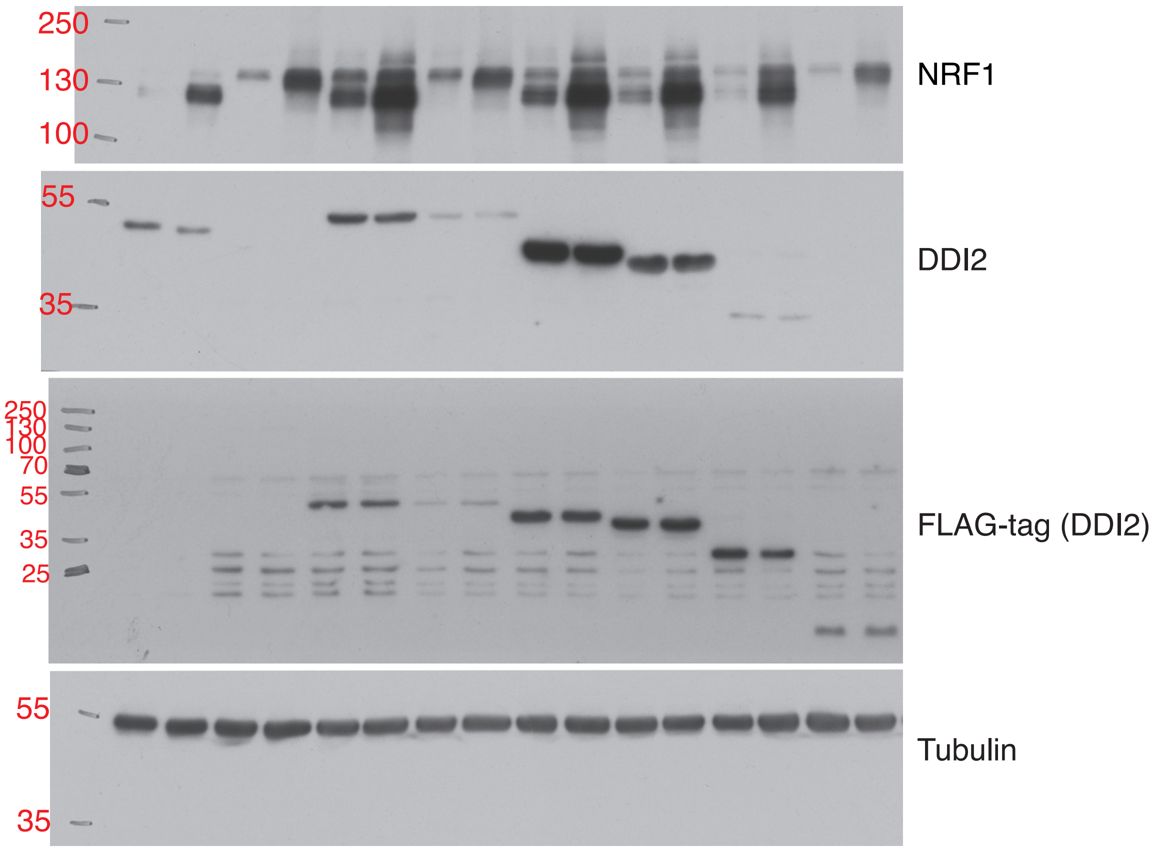

Figure 4a Replica 1

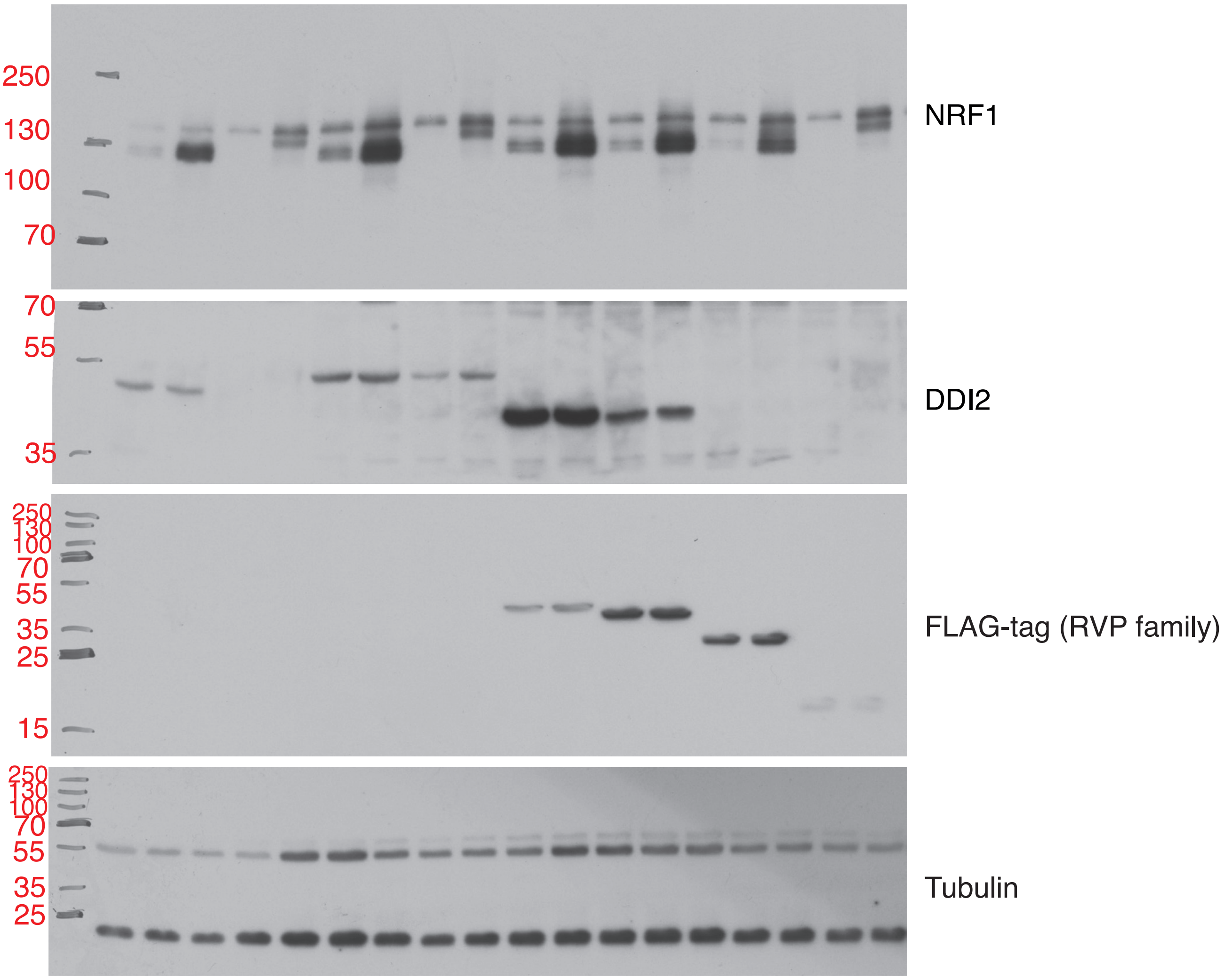

Figure 4a Replica 2

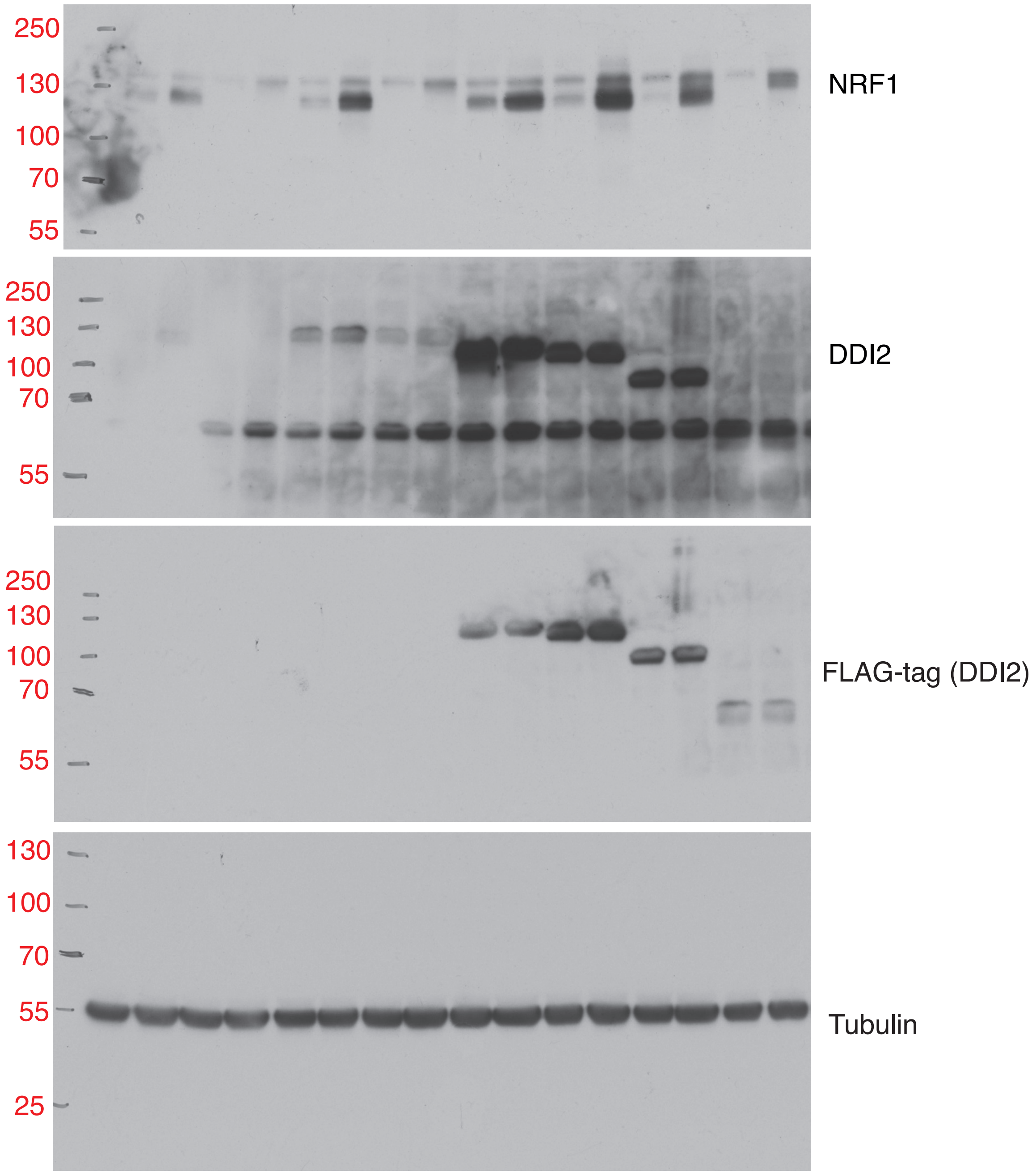

Figure 4a Replica 3

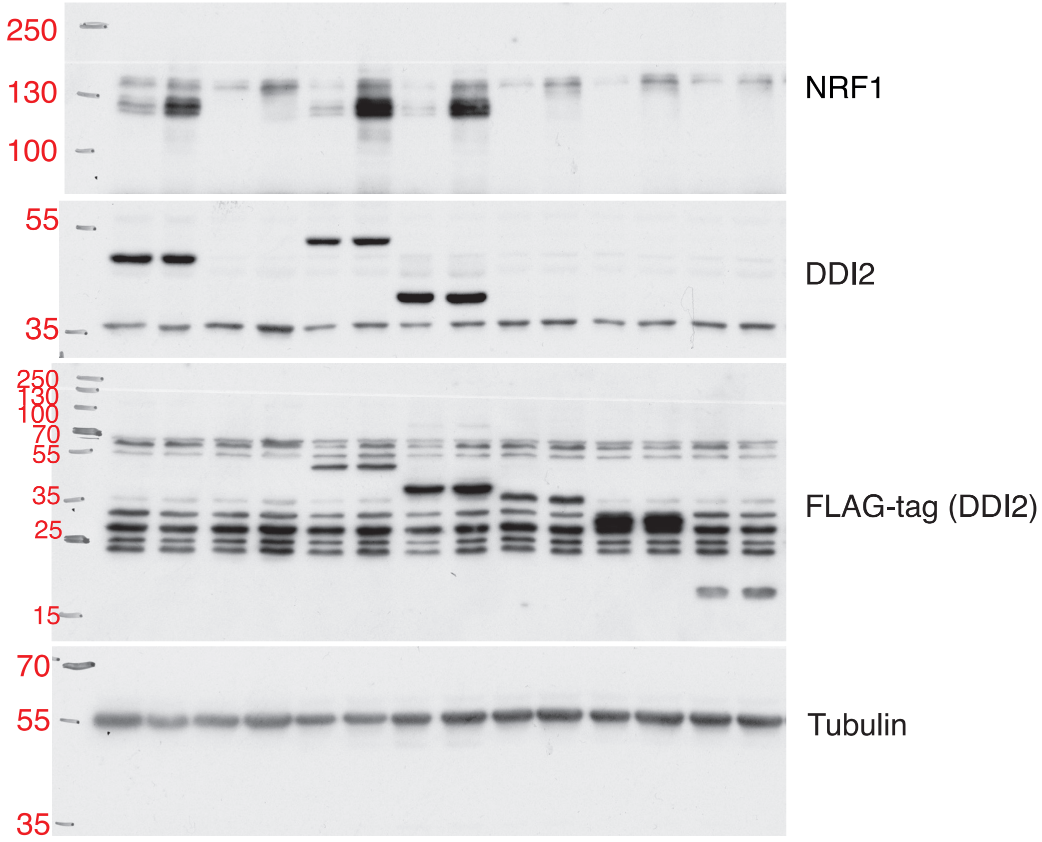

Figure 4d Replica 1

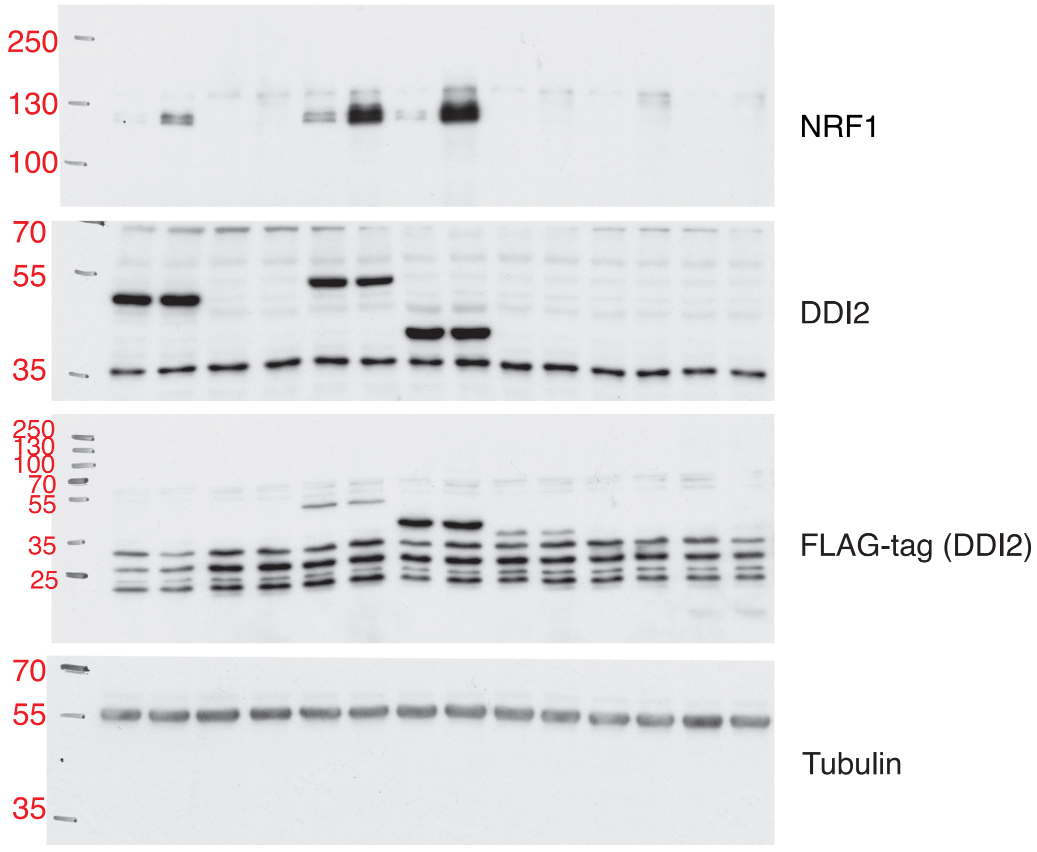

Figure 4d Replica 2

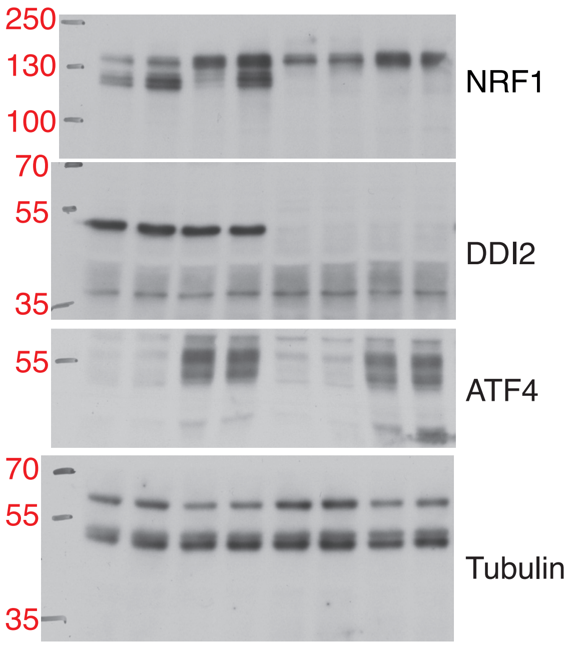

Figure 5a Replica 1

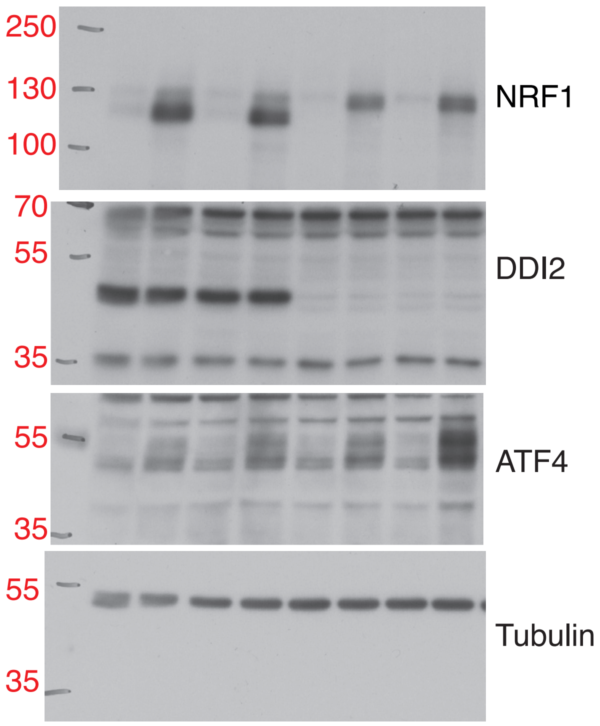

Figure 5a Replica 2

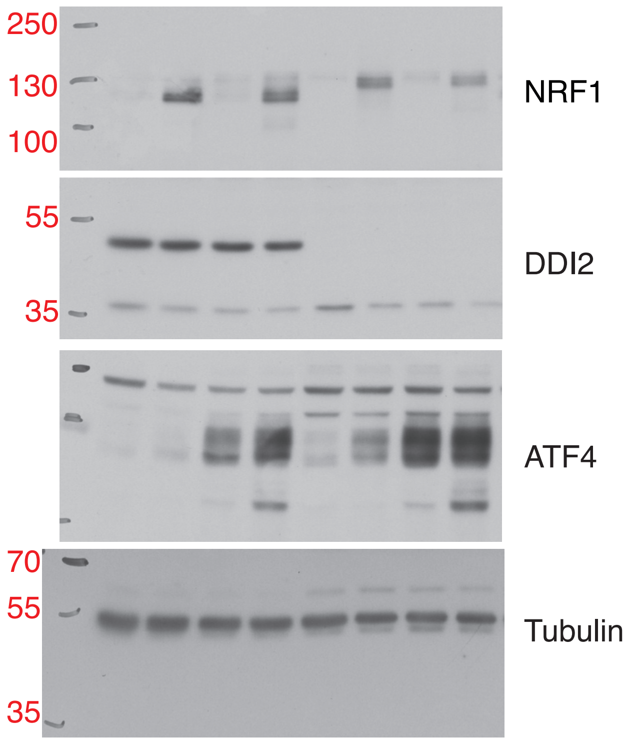

Figure 5a Replica 3

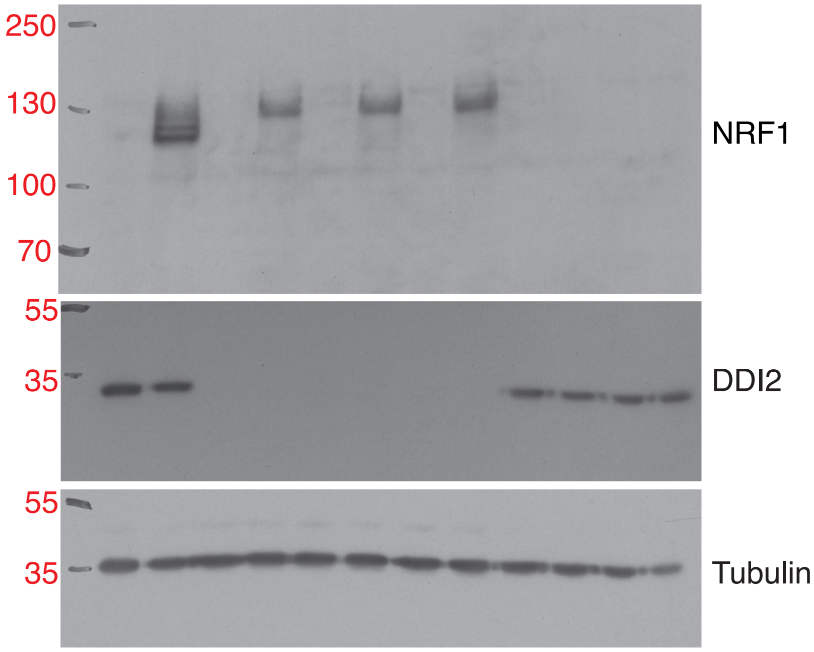

Figure s1a

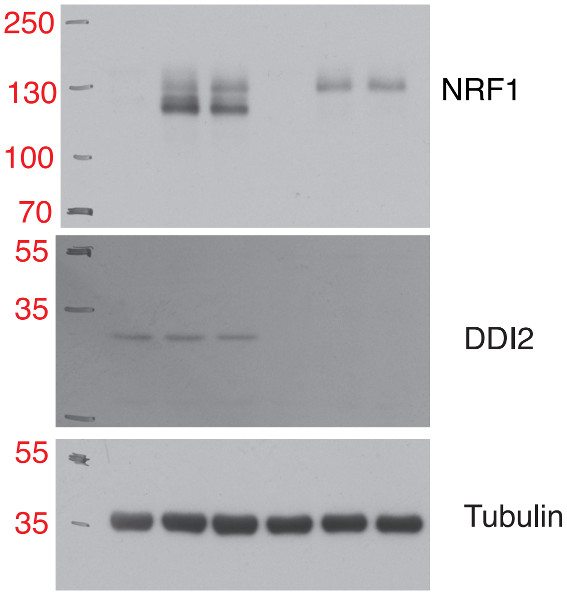

Figure s1b

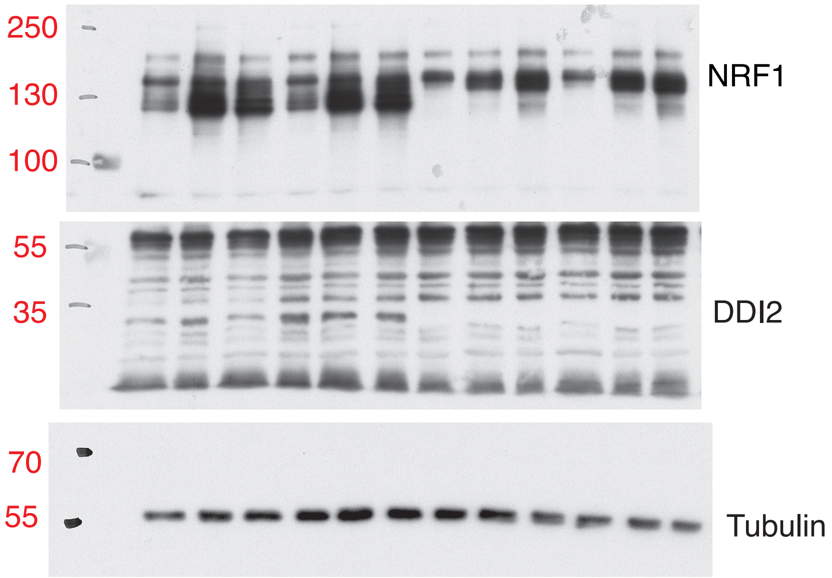

Figure s1c

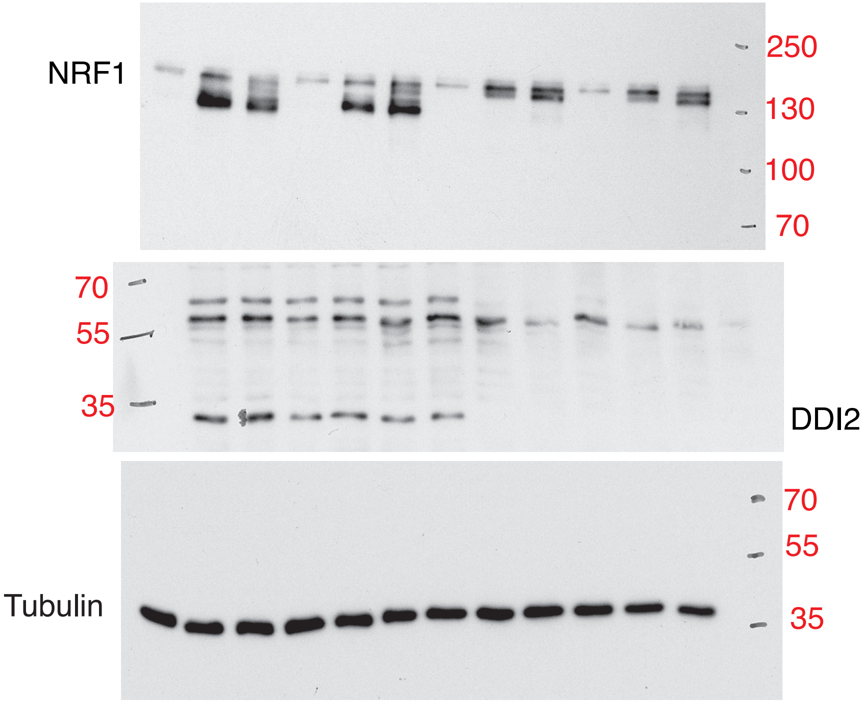

Figure s1d

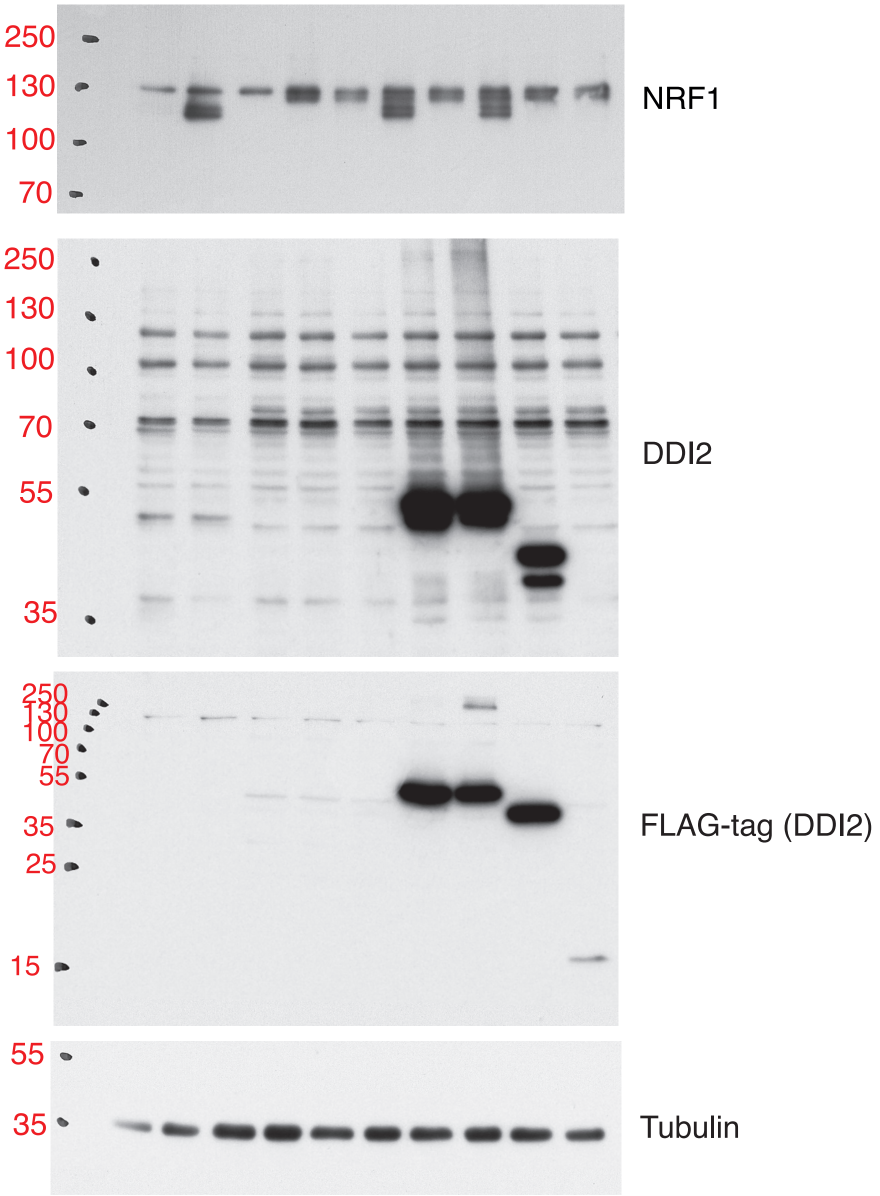
250
130
100
70
NRF1
250
130
100
70
55
35
DDI2
250
130
100
70
55
35
25
15
FLAG-tag (DDI2)
55
35
Tubulin

Figure s2

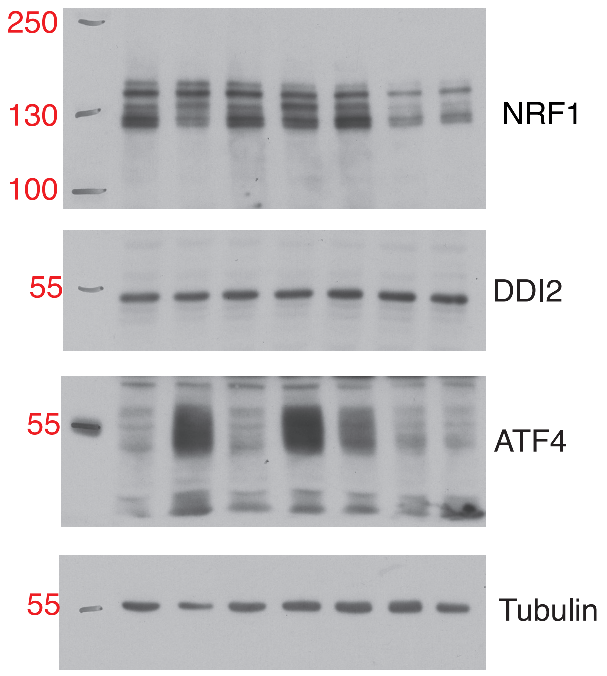

Figure s3a

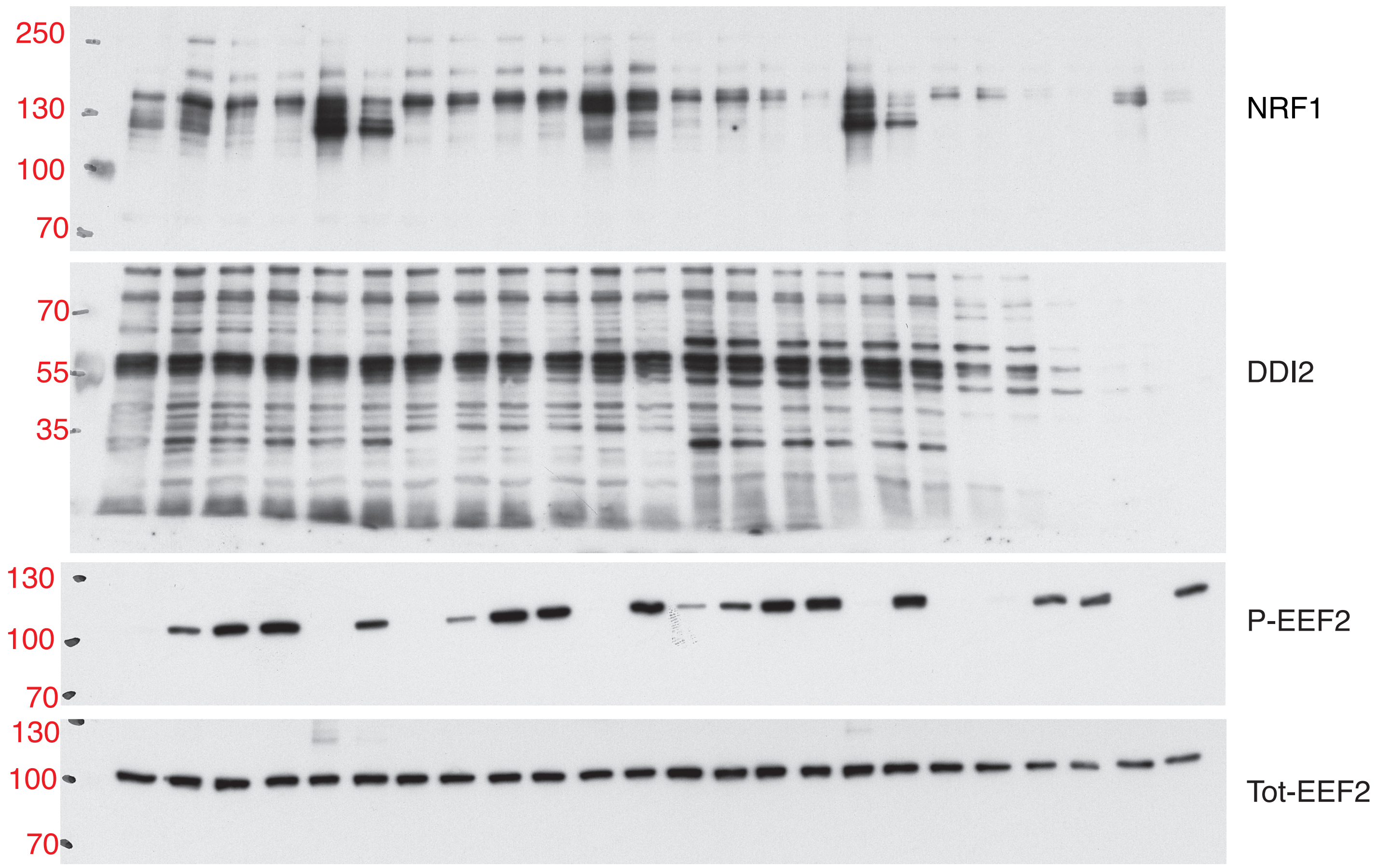

Figures s3b and s3c

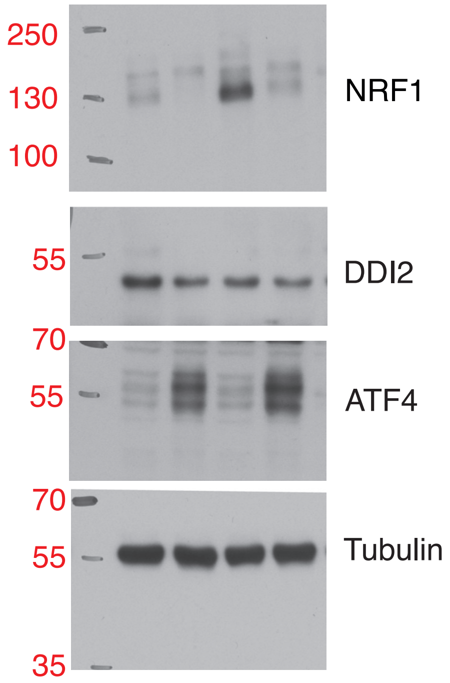

Figure s3d

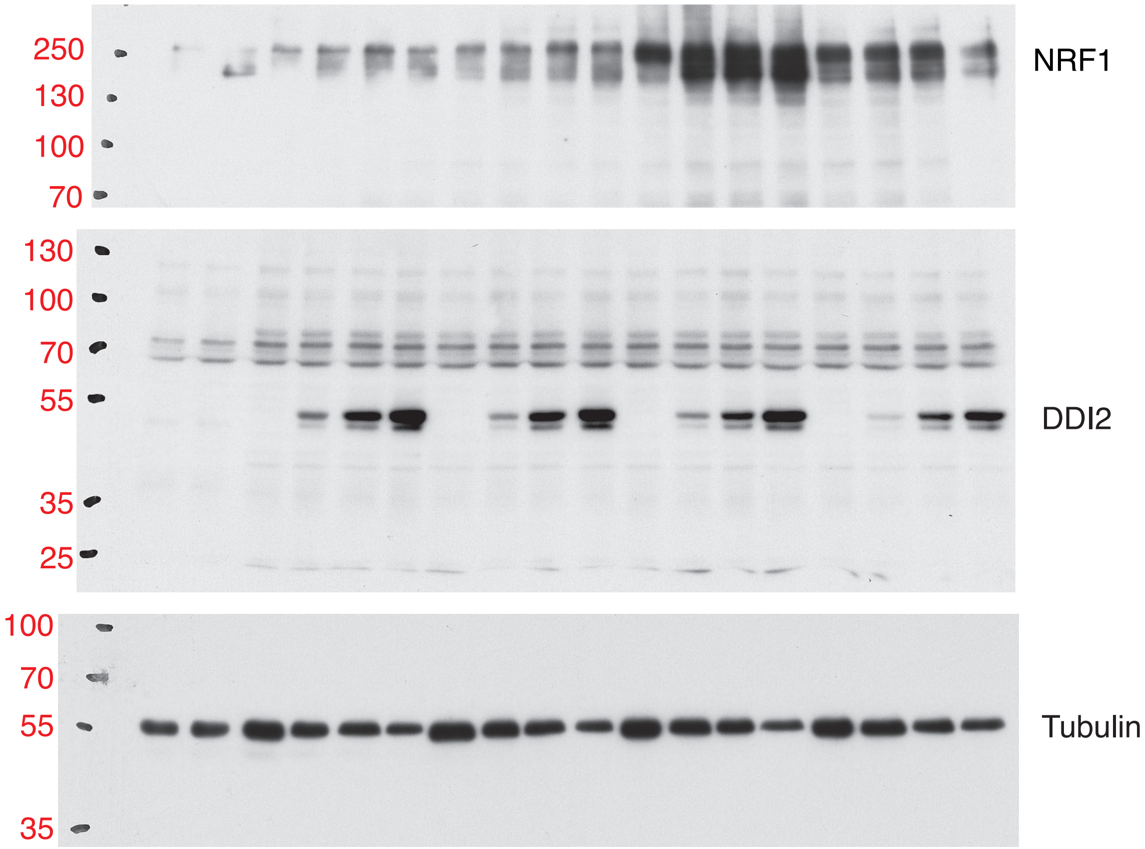

Figure s3e
